# Machine learning for the prediction of sepsis-related death: a systematic review and meta-analysis

**DOI:** 10.1186/s12911-023-02383-1

**Published:** 2023-12-11

**Authors:** Yan Zhang, Weiwei Xu, Ping Yang, An Zhang

**Affiliations:** 1 Department of Critical Care Medicine, The Second Affiliated Hospital of Chongqing Medical University, Chongqing, 400010 China; 2 Department of Endocrine and Metabolic Diseases, The Second Affiliated Hospital of Chongqing Medical University, Chongqing, 400010 China

**Keywords:** Mortality, Machine learning, Systematic review, Sepsis, Meta-analysis

## Abstract

**Background and objectives:**

Sepsis is accompanied by a considerably high risk of mortality in the short term, despite the availability of recommended mortality risk assessment tools. However, these risk assessment tools seem to have limited predictive value. With the gradual integration of machine learning into clinical practice, some researchers have attempted to employ machine learning for early mortality risk prediction in sepsis patients. Nevertheless, there is a lack of comprehensive understanding regarding the construction of predictive variables using machine learning and the value of various machine learning methods. Thus, we carried out this systematic review and meta-analysis to explore the predictive value of machine learning for sepsis-related death at different time points.

**Methods:**

PubMed, Embase, Cochrane, and Web of Science databases were searched until August 9th, 2022. The risk of bias in predictive models was assessed using the Prediction model Risk of Bias Assessment Tool (PROBAST). We also performed subgroup analysis according to time of death and type of model and summarized current predictive variables used to construct models for sepsis death prediction.

**Results:**

Fifty original studies were included, covering 104 models. The combined Concordance index (C-index), sensitivity, and specificity of machine learning models were 0.799, 0.81, and 0.80 in the training set, and 0.774, 0.71, and 0.68 in the validation set, respectively. Machine learning outperformed conventional clinical scoring tools and showed excellent C-index, sensitivity, and specificity in different subgroups. Random Forest (RF) and eXtreme Gradient Boosting (XGBoost) are the preferred machine learning models because they showed more favorable accuracy with similar modeling variables. This study found that lactate was the most frequent predictor but was seriously ignored by current clinical scoring tools.

**Conclusion:**

Machine learning methods demonstrate relatively favorable accuracy in predicting the mortality risk in sepsis patients. Given the limitations in accuracy and applicability of existing prediction scoring systems, there is an opportunity to explore updates based on existing machine learning approaches. Specifically, it is essential to develop or update more suitable mortality risk assessment tools based on the specific contexts of use, such as emergency departments, general wards, and intensive care units.

**Supplementary Information:**

The online version contains supplementary material available at 10.1186/s12911-023-02383-1..

## Introduction

Sepsis is a life-threatening organ malfunction due to the host dysregulated reaction to infection [1]. In 2017, World Health Organization (WHO) and World Health Assembly (WHA) embraced a resolution on enhancing the diagnosis, prevention, and management to decrease the burden of sepsis [2]. Due to its relatively high incidence and mortality rate, sepsis continues to be a significant public health concern [3, 4]. Existing research indicates that the in-hospital mortality rate of sepsis surpasses the average mortality rate within the same medical department, particularly in intensive care units [5, 6]. Hence, the early prediction of mortality risk in sepsis patients holds crucial clinical significance, as it can assist healthcare professionals in determining the patient’s disease status, improving treatment efficacy, and consequently reducing the risk of early mortality in patients.

Currently, there are a variety of clinical scoring systems to help clinicians assess the severity of sepsis and predict the occurrence of adverse events, such as the Simplified Acute Physiology Score II (SAPS II) [7], Acute Physiology and Chronic Health Evaluation II scoring system (APACHE II) [8], Sequential Organ Failure Assessment (SOFA) [9], and quick Sequential Organ Failure Assessment (q SOFA) [1]. However, the calibration and perception ability of these scores in predicting the risk of in-hospital death in patients with sepsis is poor [10, 11]. Moreover, these scores are set for the overall critically ill patients, rather than sepsis patients [12, 13].

In recent years, machine learnings methods have been widely used in the prediction of disease prevention, diagnosis, treatment and prognosis, such as disease risk prediction [14], patient re-admission prediction [15], and death prediction [16]. Machine learning has good predictive performance [17]. It can efficiently predict the occurrence of adverse outcomes compared with commonly used clinical scores [18–22]. The meta-analysis by Lucas M. Fleuren et al. [23] comprehensively analyzed data from 28 original studies involving 130 models. Their results demonstrate that machine learning methods exhibit relatively favorable accuracy in predicting the occurrence of sepsis. However, there is a lack of systematic evidence regarding mortality risk prediction. Therefore, we carried out this systematic review and meta-analysis to dynamically estimate the predictive value of machine learning for risk stratification of sepsis-related death and provide guidance for the development and update of sepsis death risk scoring tools.

## Methods

This systematic review was performed in line with the standards of the Preferred Reporting Items for Systematic Reviews and Meta-analyses (PRISMA 2020) statement [24]. Before the start of the study, study protocol was registered and sanctioned on the international prospective register of systematic reviews PROSPERO (reference number CRD 42022355565).

### Information sources and search strategy

For this meta-analysis, we comprehensively and systematically searched PubMed, Embase, Cochrane, and Web of Science. August 9th, 2022 was the last search date. The search method adopts the form of subject headings and free words, with no restriction on regions and languages. The search terms were designed through a combination of subject headings and free-text keywords related to sepsis, machine learning, and mortality. Afterward, we merged the search results using the ‘AND’ logical operator to assemble our definitive retrieval set. A comprehensive search strategy is provided in Table S1

### Inclusion and exclusion criteria

#### Inclusion criteria

Studies meeting the following standards were included: (1) Research subjects are patients with sepsis. (2) Research designs are cohort studies, case-control studies, case-cohort studies, and nested case-control studies. (3) The outcome event predicted by the model is mortality, and machine learning prediction models were constructed specifically focused on death-related outcomes. (4) Time of death is not limited to in-hospital death and death 28 days after discharge. (5) Studies lacking an independent validation dataset are also included in our systematic review. The term “independent validation set” denotes a situation in which the study data is segregated into a training set and a test set, or when the study encompasses a training set, a validation set, and a test set. (6) Various machine learning studies published on the same dataset. (7) Original research published in English. (8) Research that did not provide information on the time or place of death.

#### Exclusion criteria

We excluded the following studies: (1) Research types such as, Meta, review, guideline, and experts’ opinion. (2) Studies only carried out risk factor analysis with uncompleted risk model. (3) Studies lacking at least one of the following indicators related to the predication accuracy of risk model: Receiver Operator Characteristic Curve (ROC), Concordance index (C-index), sensitivity, specificity, accuracy, recovery rate, precision rate, confusion matrix, diagnostic four-grid table, F1 score, calibration curve. (4) In the clinical applications of machine learning, standardized criteria for defining small sample sizes are lacking, and EPV > 10 is required during model construction (i.e., the number of positive events, such as deaths in our study, is more than 10 times the modeling variable in the training set). Hence, in our particular context, we have defined studies with limited samples as those encompassing fewer than 50 cases. Consequently, studies with sample sizes of < 50 cases were excluded. (5) Studies primarily focused on the validation of assessment scales. (6) Research centered on the precision of single-factor predictions. (7) Case series, case reports, randomized controlled trials, and descriptive inquiries. (8) Research specifically related to pediatric populations.

### Study selection and data extraction

Retrieved literatures were imported into Endnote X9 and excluded duplicate literature. The titles and abstracts were screened to exclude ineligible studies. Full text articles were downloaded and read to include eligible studies in our systematic review. Before data extraction, we formulated a standard data extraction spreadsheet. The basic characteristics of the included studies are provided in Table S2.

Literature screening and data extraction were conducted by two independent researchers Zhang (a practicing physician specializing in critical care medicine with seven years of experience in the field) and Yang (a deputy chief physician in the department of critical care medicine with 12 years of experience). After cross-checking, disagreement was resolved by a third researcher Xu (with 20 years of experience in nursing profession), if there was any.

### Quality assessment

We performed risk of bias assessments of predictive models using Prediction model Risk of Bias Assessment Tool (PROBAST) [25], which contains many questions in four different dimensions: participants, predictors, outcomes, and statistical analysis, exhibiting overall risk of bias and overall applicability. The four dimensions contain 2, 3, 6, and 9 particular questions, respectively. Each question has three responses (yes/possibly yes, no/possibly no, and no information). A dimension is considered high risk if one of its questions is answered as no or possibly no. To be considered low risk, a dimension should have all questions answered yes or possibly yes. The overall risk of bias was rated as low risk when all dimensions were considered low risk, and as high risk when at least one dimension was considered high risk.

Two researchers (Zhang and Yang) independently conducted a risk of bias assessment based on PROBAST. After cross-checking, disagreement was resolved by a third researcher (Xu), if there was any.

### Outcome measures

The main outcome measure of our systematic review is the C-index, which reflects the overall accuracy of machine learning. In addition, this study considered that the C-index of constructed machine learning models is not enough to reflect the predictive value and actual predictive accuracy of machine learning models for sepsis-related deaths when the number of cases in the dead population and the living population is utterly unbalanced. Therefore, our primary outcome measures also included sensitivity and specificity. The secondary outcome measure for this study was the frequency of the model predictor variables.

### Data synthesis

We performed a meta-analysis on the indicators (C-index, sensitivity, and specificity) of the machine learning model. If C-index with 95% confidence interval and standard error were missing, we referred to the research of Debray TP et al. [26] to estimate the standard error. In view of the disparities in the variables included in each machine learning model and the inconsistency of parameters, we gave priority to a random-effects model for meta-analysis on C-index.

For meta-analysis for sensitivity and specificity in machine learning for mortality prediction, 2*2 tables (comprising true positives, false positives, false negatives, and true negatives) are needed. However, some included studies lacked complete 2*2 tables. Therefore, we primarily employed the following methods for estimation: (1) the 2*2 table was estimated using sensitivity, specificity, precision, and case counts. (2) Sensitivity and specificity were extracted from the ROC curve analysis based on the optimal Youden’s index to calculate the 2*2 table in conjunction with case counts.

We also performed meta-analysis of sensitivity and specificity using a bivariate mixed-effects model. The meta-analysis of this study was applied in R 4.2.0 (R development Core Team, Vienna, http://www.R-project.org). The R packages utilized for this analysis included ‘metafor,‘ ‘meta,‘ and ‘forestplot’.

## Results

### Study selection

**Fig. 1:**
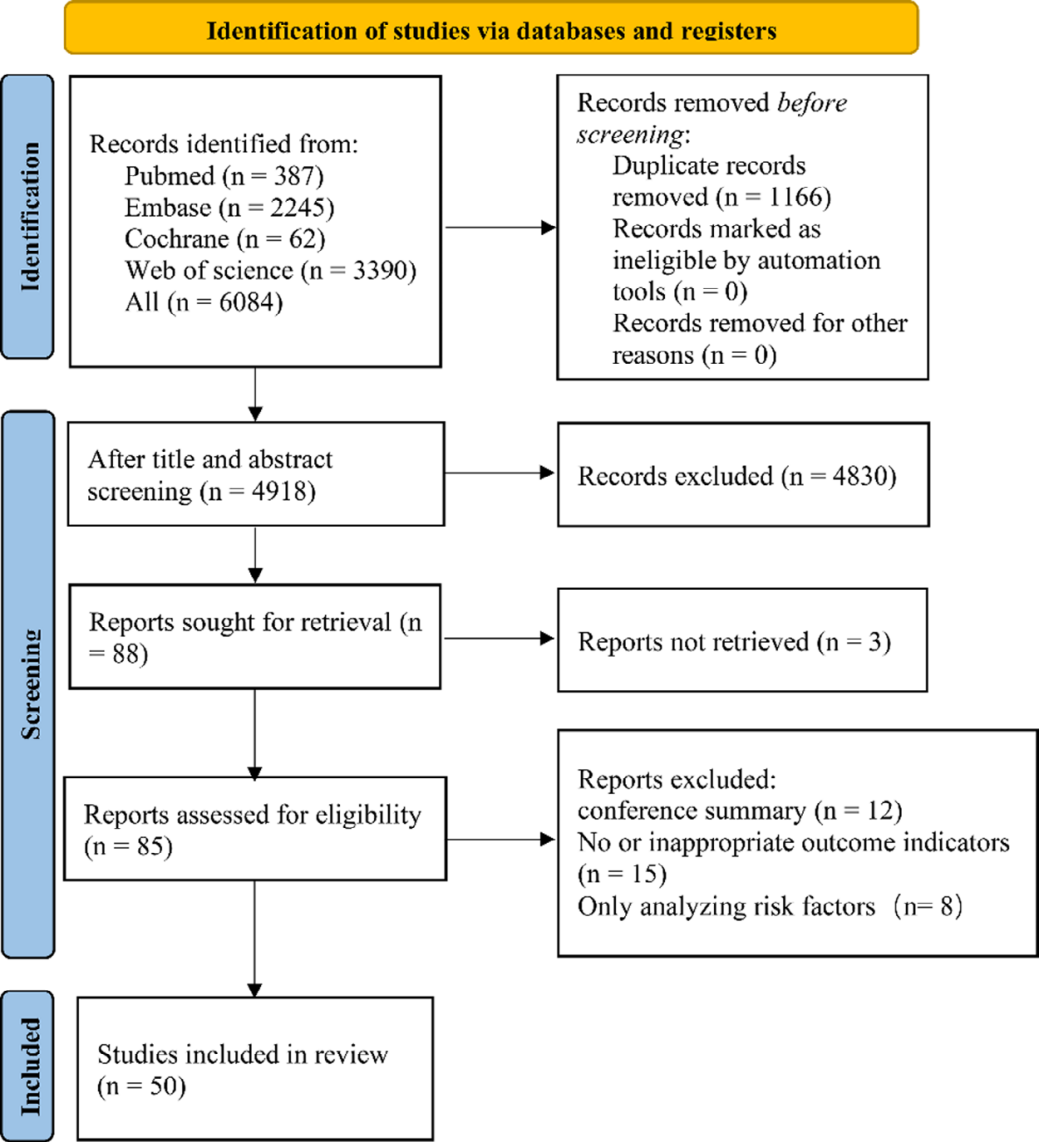
Flowchart of literature screening

A total of 6,084 articles were retrieved, and 1,166 duplicate articles were excluded. The full texts of 85 articles were downloaded and read. Conference abstracts (n = 12), studies without outcome indicators or with inappropriate outcome indicators (n = 15), and those only conducting risk factor analysis (n = 8) were deleted. Finally, 50 original studies were included. The literature screening procedure is shown in Fig. 1.

### Study characteristics

Our research included 50 original studies [22, 27–75] involving 1,928,030 patients, with 270,361 dead cases (14.02%); 247,519 died in the hospital (91.6%) and 13,739 died in January (5.1%).

This study included 104 machine learning models, including Naive Bayes (NB), Random Forest (RF), Artificial Neural Networks (ANN), eXtreme Gradient Boosting (XGBoost), Logistic Regression (LR), Decision Tree (DT), K-nearest neighbor (KNN), Survival model, Least Absolute Shrinkage and Selection Operator (LASSO), Support Vector Machine (SVM), and Blending model.

**Fig. 2:**
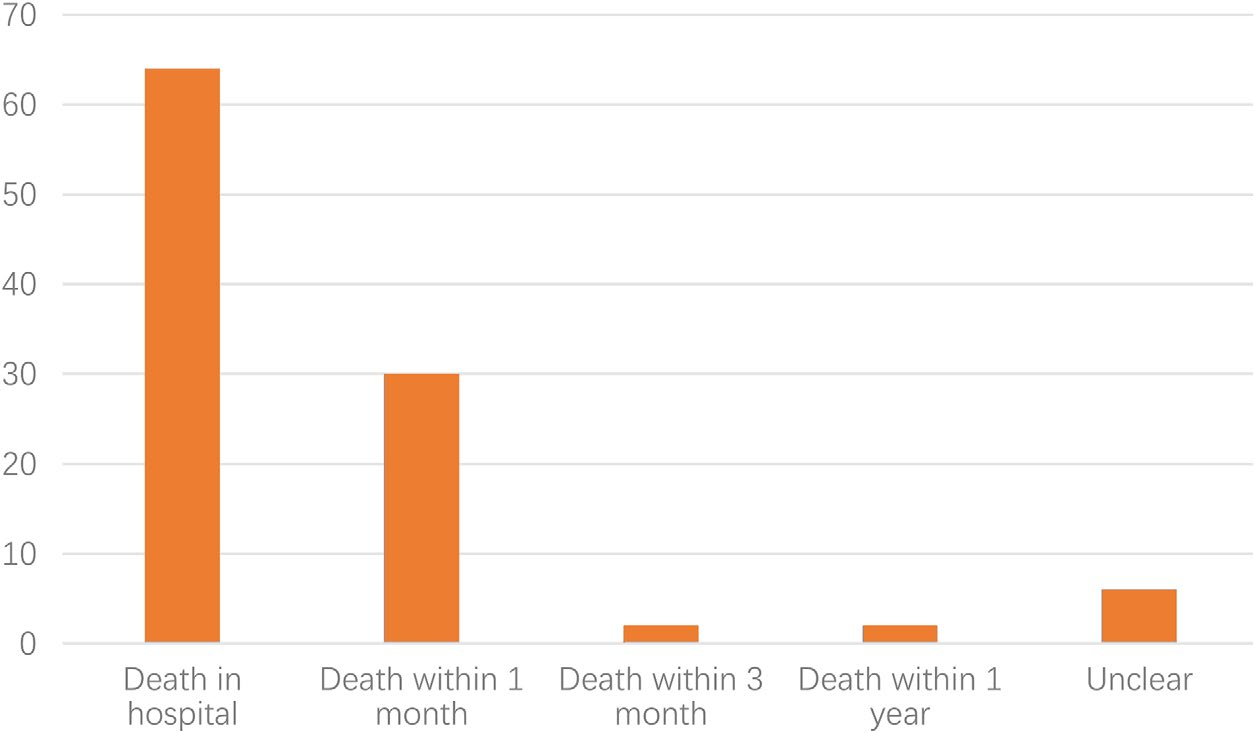
Bar chart depicting the number of models

Among these models, 25 studies built 64 machine learning models for predicting in-hospital death [22, 27, 31, 33, 35, 43, 45, 46, 48, 49, 52–55, 58, 60, 61, 63, 66, 68, 69, 71, 74, 75], 21 studies built 30 machine learning models for predicting death within 1 month [28–30, 34, 37–39, 41, 44, 47, 48, 50, 51, 55, 56, 59, 65, 68, 72, 73], two studies built two machine learning models for predicting death within 3 months [32, 71], other two studies built two machine learning models for predicting death within 1 year [62, 70], and 4 studies built 6 machine learning models that did not specifically describe the time of sepsis death [40, 42, 64, 67], these models are depicted in Fig. 2. There are relatively few research data on long-term death of sepsis. Therefore, this research mainly focused on short-term death of sepsis. **Table S2** gives an outline of key characteristics per study.

### Risk of bias in included studies

**Fig. 3:**
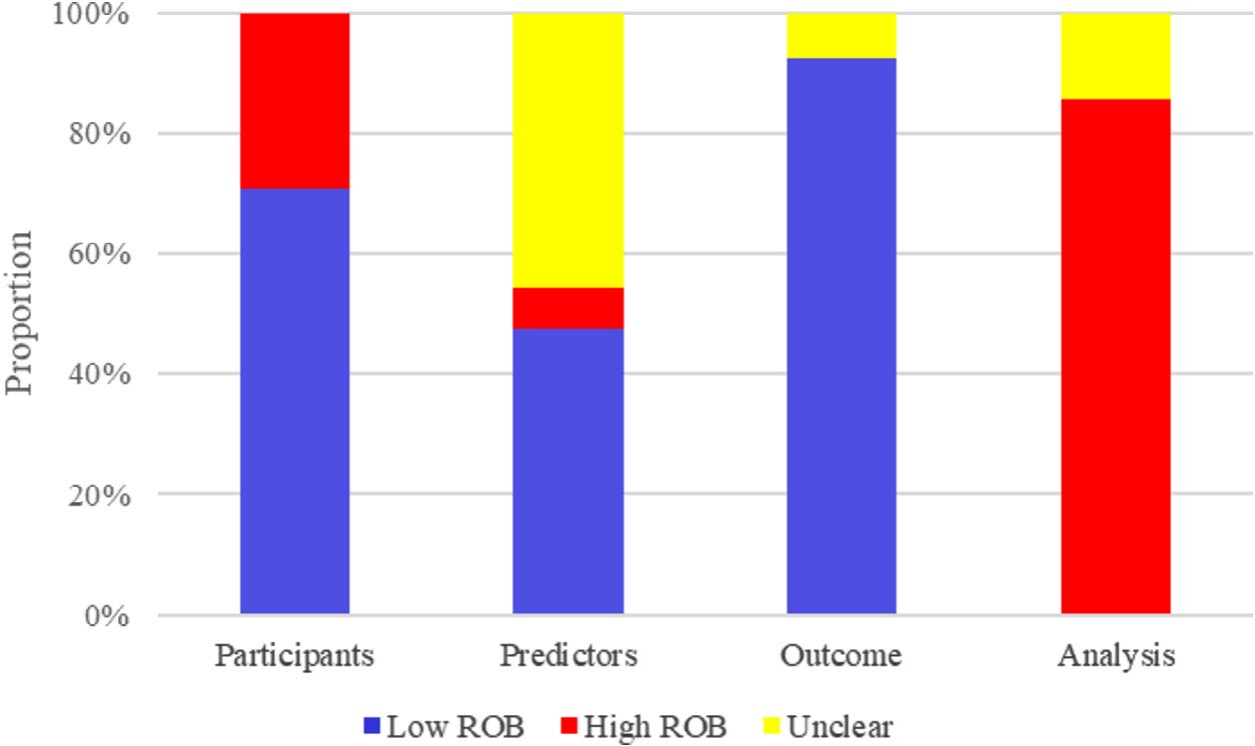
Risk of bias assessment results

The PROBAST assessment tool was used to evaluate the risk of bias of prediction model. The assessment was carried out from four aspects: predictors, participants, outcome and analysis. The results of the risk of bias assessment are shown in Fig. 3 and Table S3.

### Meta-analysis

#### C-index

According to a comprehensive analysis of data from 50 studies, the overall C-index of machine learning models predicting sepsis-related death, in the training and validation sets, was 0.799 (95%CI: 0.779–0.819) and 0.774 (95%CI: 0.763–0.785), respectively. However, the pooled overall C-index of the clinical scoring tools was 0.717 (95%CI: 0.673–0.761) and 0.689 (95%CI: 0.633–0.745), respectively. The results show that C-index of machine learning models is superior to that of clinical scoring tools for predicting sepsis-related death.

According to subgroup analysis of model types, the 104 models included in the training set were ANN (n = 11; 10.6%), DT (n = 8; 7.7%), KNN (n = 4; 3.8%), LR (n = 33; 31.7%), RF (n = 14; 13.5%), SVM (n = 14; 13.5%), XGBoost (n = 14; 13.5%), NB (n = 2; 1.9%), Survival model (n = 3; 2.9%), and LASSO (n = 1). The 88 models included in the validation set were ANN (n = 13; 14.8%), DT (n = 7; 8%), KNN (n = 4; 4.5%), LR (n = 23; 21%), RF (n = 13; 14.8%), SVM (n = 13; 14.8%), XGBoost (n = 9; 10.2%), NB (n = 3; 3.4%), Blending model (n = 1), and LASSO(n = 2; 2.3%). Almost every machine learning model had a better C-index than the clinical scoring tools (C-index: 0.717 in the training set and 0.689 in the validation set). Among the above-mentioned models, RF (C-index: 0.834 in the training set and 0.827 in the verification set) and XGboost (C-index: 0.829 in the training set and 0.805 in the verification set) had the best predictive performance, respectively. RF, in particular, showed surprisingly similar effects between the training and the validation sets, which avoided overfitting to a certain extent. Hence, RF and XGboost may be our preferred modeling schemes.

**Fig. 4:**
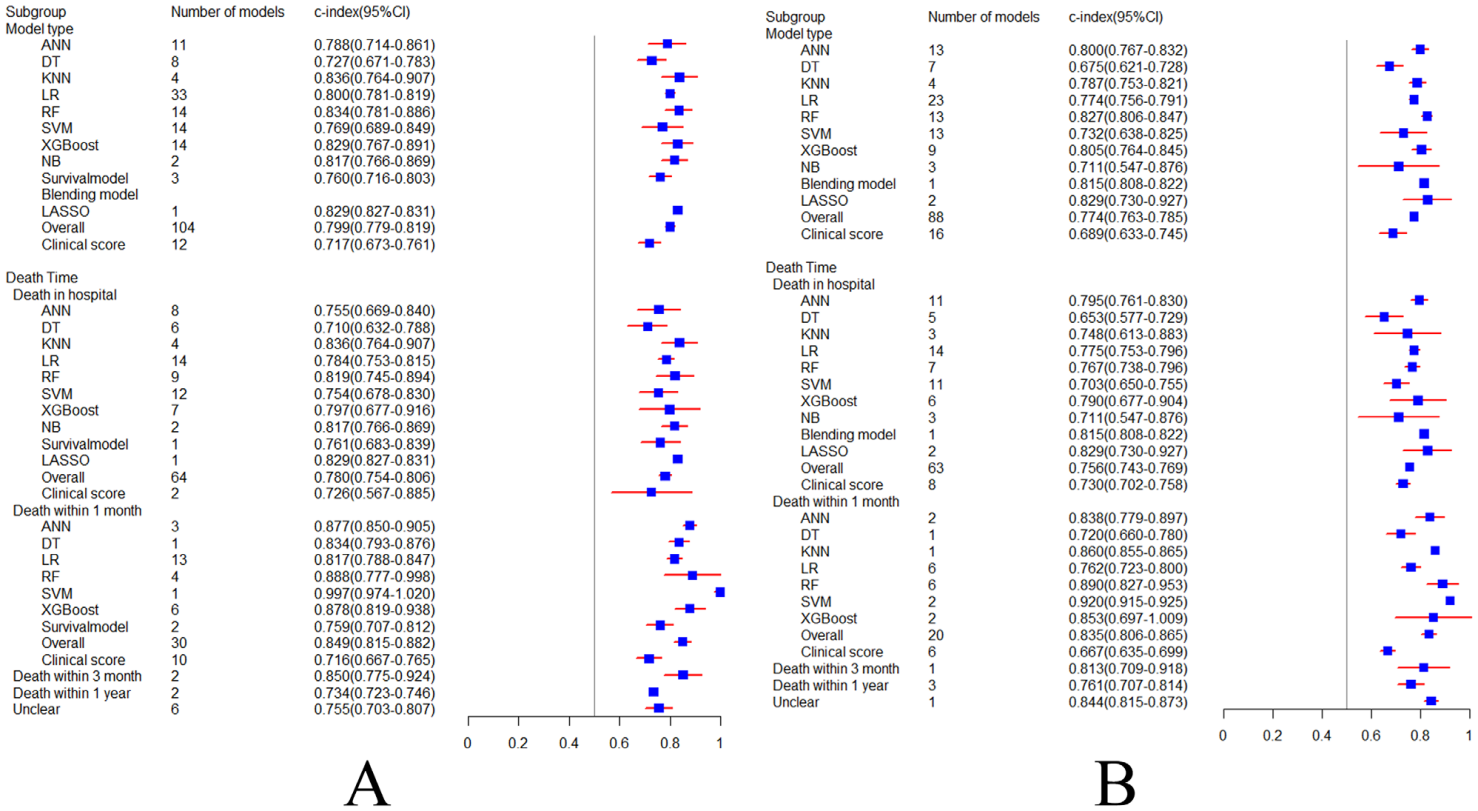
C-index forest plot of training set (**A**) and validation set (**B**)

Subgroup analysis was conducted according to time of death. In the in-hospital death subgroup, a total of 64 machine learning models were included in the training set. Its combined C-index was 0.780 (95%CI: 0.754–0.806). However, C-index of clinical scoring tools (n = 2) was 0.726 (95%CI: 0.567–0.885). A total of 63 machine learning models were included in the verification set, and their combined C-index was 0.756 (95%CI: 0.743–0.769), and C-index of clinical scoring tools (n = 8) was 0.730 (95%CI: 0.702–0.758). These results show that in the subgroup of in-hospital deaths, the C-index of the machine learning models is slightly better than clinical scoring tools. However, because the sample size of clinical scoring tools involved in this statistical analysis is too small, the results still need more verification. In 1-month death subgroup, a total of 30 machine learning models were included in the training set, and their combined C-index was 0.849 (95%CI: 0.815–0.882), and C-index of the clinical scoring tools (n = 10) was 0.716 (95%CI: 0.667–0.765). However, in the verification set a total of 20 machine learning models were included, and their combined C-index was 0.835 (95%CI: 0.806–0.865), and C-index of the clinical scoring tools (n = 6) was 0.667 (95%CI: 0.635–0.699). The results show that in the 1-month death subgroup, the C-index of machine learning models was significantly better than the clinical scoring tools. The C-index forest plot is shown in Fig. 4.

#### Sensitivity and specificity

In the training set, the combined sensitivity and specificity of the machine learning models in predicting sepsis-related death were 0.81 (95%CI: 0.75–0.86) and 0.80 (95%CI: 0.72–0.86), respectively. However, the combined sensitivity and specificity of the clinical scoring tools in predicting sepsis-related death were 0.65 (95%CI: 0.61–0.68) and 0.68 (95%CI: 0.65–0.71), respectively.

In the validation set, the combined sensitivity and specificity of the machine learning models in predicting sepsis-related death were 0.71 (95%CI: 0.67–0.74) and 0.68 (95%CI: 0.59–0.77), respectively. However, the combined sensitivity and specificity of the clinical scoring tools in predicting sepsis-related death were 0.52 (95%CI: 0.42–0.62) and 0.28 (95%CI: 0.04–0.78), respectively. The results suggest that machine learning models are better than conventional clinical scoring tools in predicting sepsis death.

According to the subgroup analysis of model types, the 65 models included in the training set were ANN (n = 5; 7.7%), DT (n = 6; 9.2%), KNN (n = 2; 3%), LR (n = 23; 35.4%), RF (n = 10; 15.4%), SVM (n = 5; 7.7%), XGBoost (n = 11; 17%), NB (n = 1), Survival model (n = 1), and LASSO (n = 1). The 67 models included in the validation set were ANN (n = 11; 16.4%), DT (n = 4; 6%), KNN (n = 4; 6%), LR (n = 17; 25.4%), RF (n = 9; 13.4%), SVM (n = 9; 13.4%), XGBoost (n = 7; 10.4%), NB (n = 3; 4.5%), and LASSO (n = 3; 4.5%). The sensitivity and specificity of machine learning models are superior to clinical scoring tools (sensitivity and specificity: training set 0.65 and 0.68, validation set 0.52 and 0.28), respectively. RF (sensitivity and specificity: training set 0.90 and 0.87, verification set 0.74 and 0.77) and XGboost (sensitivity and specificity: training set 0.83 and 0.86, verification set 0.68 and 0.75) had the fittest predictive performance in both training and validation sets.

Subgroup analysis was carried out according to time of death. In the in-hospital death subgroup, a total of 37 machine learning models were included in the training set. The combined sensitivity and specificity were 0.85 (95%CI: 0.75–0.92) and 0.86 (95%CI: 0.73–0.93), respectively. However, the sensitivity and specificity of the clinical scoring tools (n = 1) were 0.59 and 0.66 respectively.

A total of 51 machine learning models were included in the verification set, and the combined sensitivity and specificity were 0.68 (95%CI: 0.64–0.72) and 0.67 (95%CI: 0.54–0.78), respectively. However, the sensitivity and specificity of the clinical scoring tools (n = 8) were 0.47 (95%CI: 0.33–0.61) and 0.10 (95%CI: 0.00-0.83), respectively. These results suggest that sensitivity and specificity of machine learning models are higher than clinical scoring tools in the subgroup of in-hospital deaths.

In the 1-month death subgroup, a total of 20 machine learning models were included in the training set, and their combined sensitivity and specificity were 0.78 (95%CI: 0.75–0.80) and 0.70 (95%CI: 0.63–0.75), respectively, while sensitivity and specificity of clinical scoring tools (n = 10) were 0.65 (95%CI: 0.62–0.69) and 0.68 (95%CI: 0.65–0.72), respectively. A total of 14 machine learning models were included in the validation set, and their combined sensitivity and specificity were 0.78 (95%CI: 0.73–0.82) and 0.75 (95%CI: 0.67–0.82), respectively. However, the sensitivity and specificity of clinical scoring tools (n = 5) were 0.62 (95%CI: 0.58–0.65) and 0.65 (95%CI: 0.58–0.72), respectively.

**Fig. 5:**
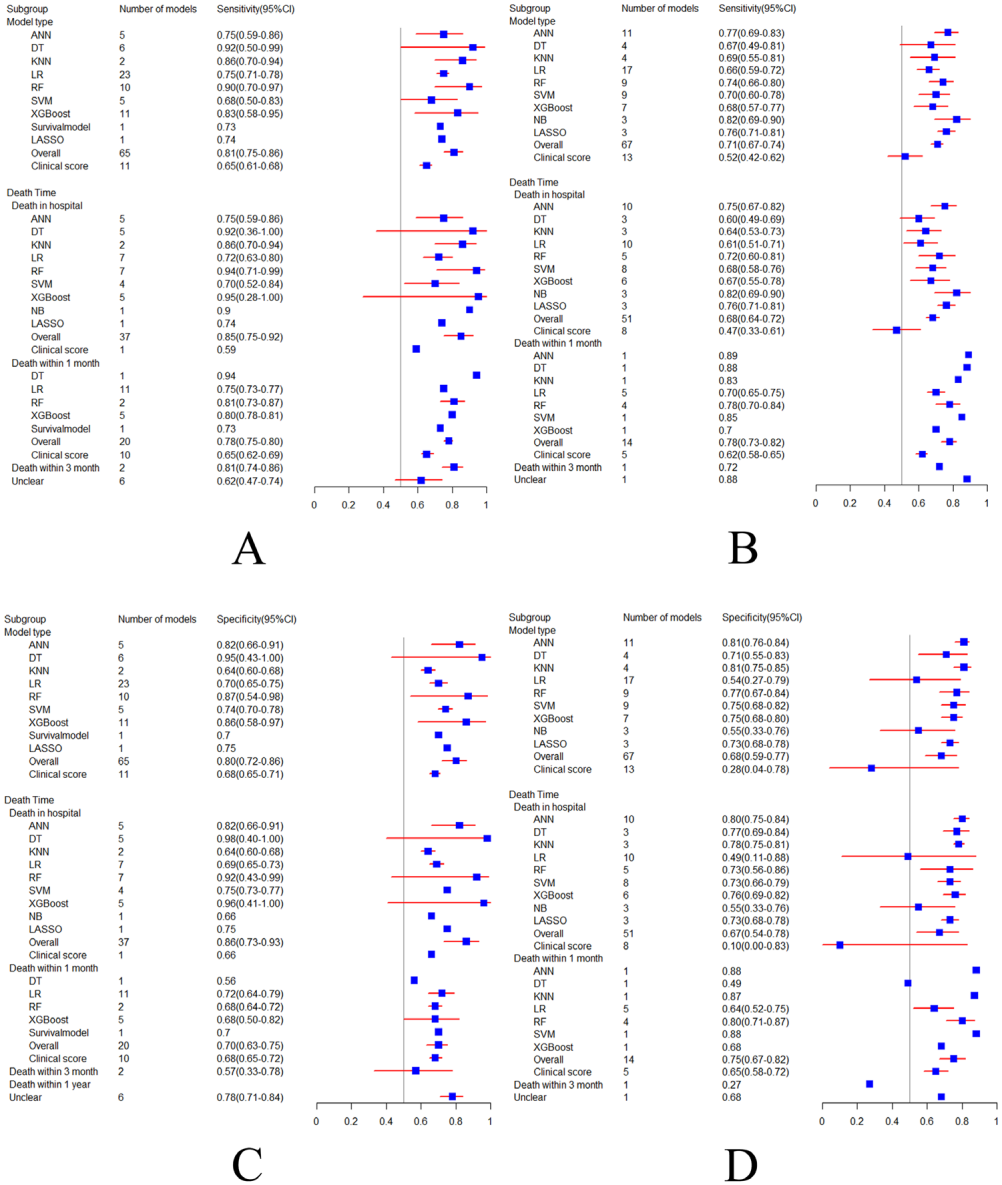
Sensitivity (**A-B**) and specificity (**C-D**) forest plot of training set (**A**, **C**) and validation set (**B**, **D**)

The results show that in the 1-month death subgroup, sensitivity and specificity of machine learning models are higher than clinical scoring tools. The sensitivity and specificity forest plot are shown in Fig. 5.

#### Modeling variables

This study summarized 125 modeling variables, and the main 30 modeling variables were: lactate, age, GCS, ventilator, systolic blood pressure, pH, heartrate, respiratory rate, temperature, gender, SpO2, SOFA score, BUN, creatinine, PLT, PaO2, INR, PCO2, urine output, shock, comorbidities, cancer, PTT, WBC, MAP, albumin, BMI, BE, race, and bicarbonate. Incorporating these frequently occurring modeling variables into machine learning models may help improve the prediction of sepsis-related death. In addition, some variables with lower frequency still need to be considered, such as: Surviving Sepsis Campaign Bundles, ScvO2, BNP, TnT, PCT, IL-6, and administration time of appropriate antimicrobial therapy.

In RF model, the modeling variables with higher frequency were age, lactate, GCS, systolic blood pressure, heartrate, respiratory rate, ventilator, pH, temperature, SpO2, BUN, creatinine, and MAP. In XGboost model, the modeling variables with higher frequency were age, lactate, systolic blood pressure, heartrate, temperature, SpO2, GCS, ventilator, BUN, creatinine, and PLT. The modeling variables with higher frequency in the two dominant models are highly similar and of great significance in clinical practice. If the two models are combined reasonably, the predictive value of RF and XGboost models for sepsis-related death may further increase on the existing basis. Modeling variables for the included models are provided in Table S4.

## Discussion

In this study, the data of 50 original studies were comprehensively analyzed, and we found that in the training and validation sets, the combined C-index, sensitivity and specificity of machine learning models in predicting sepsis-related death were higher than clinical scoring tools. The results of this study are in line with those of Raith et al. [76]. The predictive models for mortality risk achieved a pooled C-index of 0.799, sensitivity of 0.81, and specificity of 0.80 in the training set, and a pooled C-index of 0.774, sensitivity of 0.71, and specificity of 0.68 in the validation set, indicating that machine learning methods demonstrate relatively favorable predictive performance for early mortality in sepsis, with no evidence of overfitting. Moreover, among the 104 machine learning models included in this study, RF and XGboost showed better predictive performance. Regarding clinical scoring tools for predicting sepsis-related death in the training set, the combined C-index, sensitivity, and specificity were 0.717, 0.65, and 0.68, respectively, in the validation set the above-mentioned values were 0.689, 0.52, and 0.28, respectively, revealing that clinical risk scoring still possesses significant limitations in predicting mortality risk in sepsis. Our research indicates that the prediction of the risk of mortality in sepsis extends beyond focusing solely on in-hospital deaths in current clinical practice, as there is a growing interest in studying out-of-hospital deaths as well. Machine learning models appear to exhibit favorable accuracy in early prediction of both in-hospital and out-of-hospital deaths. In-hospital mortality is primarily concentrated in the ICU and emergency department. Therefore, we believe that the development and update of sepsis-specific mortality risk assessment tools tailored specifically for intensive care units and emergency departments are imperative. This would enable clinicians to promptly formulate effective diagnostic and treatment decisions, thereby reducing the risk of mortality. Additionally, effective predictive tools should be developed for assessing the risk of out-of-hospital mortality to provide support to patients’ families and the community, with the aim of improving patient quality of life and potentially extending their survival time.

Additionally, we also conducted a meta-analysis of 38 studies on the accuracy of SOFA score and qSOFA score in predicting short-term mortality risk in sepsis patients. The results of our study show that the predictive value of SOFA score for sepsis-related mortality remains unsatisfactory, which is consistent with those of Fernando et al. [13]. Their review reported low sensitivity and specificity values for both SOFA and qSOFA scores in predicting the risk of death in sepsis patients (sensitivity values were 0.685 and 0.608, and specificity values were 0.688 and 0.595, respectively).

For machine learning models, the selection of appropriate modeling variables is a key factor in improving their predictive accuracy, some studies have found that blood lactic acid could be treated as a predictor of mortality in patients with sepsis, and combining blood lactic acid with the existing scores could increase the predictive accuracy of the scoring system [12, 77]. Lactic acid is an independent predictor of death in patients with septic shock; mortality rate of patients can increase with the elevation of lactic acid level [78–80]. Lactic acid not only predicts high risk of death but can also guide sepsis treatment [81, 82]. However, almost none of the existing scores included this indicator. In this study, when calculating the frequency of statistical modeling variables, we found that the frequency of lactic acid was the highest, suggesting that lactic acid can be used as an important variable for predicting sepsis death and an important predictor in the advance of simple risk assessment tools for clinical visualization in the future. At the same time, we found that the main 30 important modeling variables involved coagulation system, respiratory system, circulatory system, nervous system, liver, kidney; multiple organ failures were closely associated with the prognosis of sepsis. It is suggested that these modeling variables are consistent with the clinical indicators for predicting the prognosis of sepsis. Creatinine and blood urea nitrogen are important indicators for evaluating renal function. Therefore, measuring serum creatinine and blood urea nitrogen in the early course of sepsis can help evaluate renal function, identify sepsis-related acute kidney injury, and predict disease progression and prognosis [83, 84]. Respiratory rate, blood pressure, and blood oxygen saturation are important health indicators of the human body, which can evaluate the function of the respiratory and circulatory systems, and are also important monitoring indicators in early resuscitation programs [85].

The modeling variables included in machine learning have clinical significance and theoretical support, with application value in predicting risk of death in patients with sepsis. In addition, this study found many variables that are ignored clinically, such as Surviving Sepsis Campaign Bundles, ScvO2, BNP, TnT, PCT, IL-6, and administration time of appropriate antimicrobial therapy. However, all these predictive models have a certain predictive value. As a result, these variables should be paid attention to in the subsequent development of scoring systems. In the process of meta-analysis, machine learning models generally have a high risk of bias in the modeling process, possibly because of the fact that bias risk assessment of predictive model is relatively critical, especially in statistical analysis. Most of the models did not meet a low risk of bias in statistical analysis, causing an excessive high proportion of bias, which needs to be dealt with to improve machine learning performance.

This study is a large-scale systematic review, which is one of it is strength points. The results show that machine learning has good predictive value for sepsis-related death and outperforms clinical scoring tools. The meta-analysis published by Lucas M. Fleuren et al. [23] showed that machine learning could precisely predict the onset of sepsis in advance, but their systematic review did not conduct a comprehensive analysis of the predictive value of model learning for sepsis-related death. However, our study fills in the gap of its predictive value for sepsis-related death. Therefore, this study can act as a theoretical support for the improvement of clinical scoring tools. By summarizing the frequency of modeling variables, we found that lactate can be used as an important predictor for the development of simple clinical visualization risk assessment tools. Numerous studies have ignored and screened out various variables, which are also of a great value for enhancing scoring system performance.

This study has some limitations as follow: (1) The identification of cases of septicemia is highly challenging and often inaccurate, particularly when relying on routine data or ICD code-based approaches. (2) The included machine learning models exhibited great risk of bias due to the obvious criticality of risk of bias assessment of predictive models. (3) Most of the included studies explored the short-term death of sepsis, such as in-hospital death and one-month death, but there are few original studies on medium and long-term death time, such as 3 months, 6 months or even one year. Therefore, the predictive value of machine learning models for long-term death time of sepsis is still lack of evidence-based medical evidence. (4) In our study, we included full-text conference papers, while other gray literature sources were not incorporated into our research. We will continue to pay attention to this field in future research. (5) In certain validation sets within the original studies, there were fewer than four models. It is important to note that the utilization of at least four models is a prerequisite when calculating positive and negative predictive values through a bivariate mixed-effects model. Consequently, this study exclusively focused on evaluating the c-index, sensitivity, and specificity. (6) Furthermore, it is worth acknowledging that some clinically relevant variables may pose challenges in terms of measurement, particularly in settings beyond the purview of major hospitals. Moreover, the accessibility of these variables for patients residing at a considerable distance from the hospital can present significant obstacles.

## Conclusion

The predictive value of clinical scoring tools is controversial and needs further improvement. Machine learning has an ideal predictive value for sepsis-related death, outperforms clinical scoring tools, and can be utilized as a predictive tool for early risk stratification. Therefore, simple scoring tools or risk equations suitable for different races are desired to be developed based on large-scale machine learning models with large samples, and cross-races.

### Electronic supplementary material

Below is the link to the electronic supplementary material.

**Supplementary Material 1**: **Table S1** Search strategy. **Table S2 **Overview of key characteristics per study. **Table S3 **Risk of bias assessment results. **Table S4 **Modeling variables

## Data Availability

The datasets used and/or analyzed during the current study are available from the corresponding author on reasonable request.
