# Supplementary material for: Machine learning for the prediction of sepsis-related death: a systematic review and meta-analysis
Source: BMC Med Inform Decis Mak. 2023 Dec 11;23:283. doi: 10.1186/s12911-023-02383-1 (PMC10712076; doi:10.1186/s12911-023-02383-1)
Supplement: Supplementary file 1 — Supplementary Material 1: Table S1 Search strategy. Table S2 Overview of key characteristics per study. Table S3 Risk of bias assessment results. Table S4 Modeling variables [file 12911_2023_2383_MOESM1_ESM.docx]

**Table S1** Literature search strategy.

**1.Pubmed**

| Search number | Query | Results |
| --- | --- | --- |
| #1 | "Sepsis"[Mesh] | 136,801 |
| #2 | ((((((((((((((((((Bloodstream Infection[Title/Abstract]) OR (Bloodstream Infections[Title/Abstract])) OR (Infection, Bloodstream[Title/Abstract])) OR (Pyemia[Title/Abstract])) OR (Pyemias[Title/Abstract])) OR (Pyohemia[Title/Abstract])) OR (Pyohemias[Title/Abstract])) OR (Pyaemia[Title/Abstract])) OR (Septicemia[Title/Abstract])) OR (Septicemias[Title/Abstract])) OR (Poisoning, Blood[Title/Abstract])) OR (Blood Poisoning[Title/Abstract])) OR (Blood Poisonings[Title/Abstract])) OR (Poisonings, Blood[Title/Abstract])) OR (septic disease[Title/Abstract])) OR (septic shock[Title/Abstract])) OR (sepsis-associated hypotension[Title/Abstract])) OR (Shock, Septic[Title/Abstract])) OR (Septic[Title/Abstract]) | 116,549 |
| #3 | ("Sepsis"[Mesh]) OR (((((((((((((((((((Bloodstream Infection[Title/Abstract]) OR (Bloodstream Infections[Title/Abstract])) OR (Infection, Bloodstream[Title/Abstract])) OR (Pyemia[Title/Abstract])) OR (Pyemias[Title/Abstract])) OR (Pyohemia[Title/Abstract])) OR (Pyohemias[Title/Abstract])) OR (Pyaemia[Title/Abstract])) OR (Septicemia[Title/Abstract])) OR (Septicemias[Title/Abstract])) OR (Poisoning, Blood[Title/Abstract])) OR (Blood Poisoning[Title/Abstract])) OR (Blood Poisonings[Title/Abstract])) OR (Poisonings, Blood[Title/Abstract])) OR (septic disease[Title/Abstract])) OR (septic shock[Title/Abstract])) OR (sepsis-associated hypotension[Title/Abstract])) OR (Shock, Septic[Title/Abstract])) OR (Septic[Title/Abstract])) | 212,961 |
| #4 | "Machine Learning"[Mesh] | 47,375 |
| #5 | ((((((((((((((((((((((((((Transfer Learning[Title/Abstract]) OR (Deep learning[Title/Abstract])) OR (Learning, Transfer[Title/Abstract])) OR (Ensemble Learning[Title/Abstract])) OR (artificial intelligence[Title/Abstract])) OR (Prediction model[Title/Abstract])) OR (random forest[Title/Abstract])) OR (artificial neural network[Title/Abstract])) OR (ANN[Title/Abstract])) OR (Support vector machine[Title/Abstract])) OR (SVM[Title/Abstract])) OR (Gradient Boosting Machine[Title/Abstract])) OR (GBM[Title/Abstract])) OR (Nomogram[Title/Abstract])) OR (XGboost[Title/Abstract])) OR (Decision tree[Title/Abstract])) OR (Development[Title/Abstract] AND validation[Title/Abstract])) OR (Risk Prediction[Title/Abstract])) OR (Risk-Prediction[Title/Abstract])) OR (machine intelligence[Title/Abstract])) OR (bayesian[Title/Abstract])) OR (naïve bayes[Title/Abstract])) OR (decision support[Title/Abstract])) OR (adaboost[Title/Abstract])) OR (Learning, Machine[Title/Abstract])) OR (learning machine[Title/Abstract])) OR (learning machines[Title/Abstract]) | 302,209 |
| #6 | ("Machine Learning"[Mesh]) OR (((((((((((((((((((((((((((Transfer Learning[Title/Abstract]) OR (Deep learning[Title/Abstract])) OR (Learning, Transfer[Title/Abstract])) OR (Ensemble Learning[Title/Abstract])) OR (artificial intelligence[Title/Abstract])) OR (Prediction model[Title/Abstract])) OR (random forest[Title/Abstract])) OR (artificial neural network[Title/Abstract])) OR (ANN[Title/Abstract])) OR (Support vector machine[Title/Abstract])) OR (SVM[Title/Abstract])) OR (Gradient Boosting Machine[Title/Abstract])) OR (GBM[Title/Abstract])) OR (Nomogram[Title/Abstract])) OR (XGboost[Title/Abstract])) OR (Decision tree[Title/Abstract])) OR (Development[Title/Abstract] AND validation[Title/Abstract])) OR (Risk Prediction[Title/Abstract])) OR (Risk-Prediction[Title/Abstract])) OR (machine intelligence[Title/Abstract])) OR (bayesian[Title/Abstract])) OR (naïve bayes[Title/Abstract])) OR (decision support[Title/Abstract])) OR (adaboost[Title/Abstract])) OR (Learning, Machine[Title/Abstract])) OR (learning machine[Title/Abstract])) OR (learning machines[Title/Abstract])) | 319,084 |
| #7 | "Mortality"[Mesh] | 419,114 |
| #8 | ((((((((Mortalities[Title/Abstract]) OR (Case Fatality Rate[Title/Abstract])) OR (Case Fatality Rates[Title/Abstract])) OR (Rate, Case Fatality[Title/Abstract])) OR (Rates, Case Fatality[Title/Abstract])) OR (CFR Case Fatality Rate[Title/Abstract])) OR (mors[Title/Abstract])) OR (death[Title/Abstract])) OR (survival[Title/Abstract]) | 1,805,939 |
| #9 | ("Mortality"[Mesh]) OR (((((((((Mortalities[Title/Abstract]) OR (Case Fatality Rate[Title/Abstract])) OR (Case Fatality Rates[Title/Abstract])) OR (Rate, Case Fatality[Title/Abstract])) OR (Rates, Case Fatality[Title/Abstract])) OR (CFR Case Fatality Rate[Title/Abstract])) OR (mors[Title/Abstract])) OR (death[Title/Abstract])) OR (survival[Title/Abstract])) | 2,016,910 |
| #10 | #3 AND #6 AND #9 | 387 |

**2.Cochrane**

| Search number | Query | Results |
| --- | --- | --- |
| #1 | MeSH descriptor: [Sepsis] explode all trees | 4960 |
| #2 | (Bloodstream Infection):ab,ti,kw OR (Bloodstream Infections):ab,ti,kw OR (Infection, Bloodstream):ab,ti,kw OR (Pyemia):ab,ti,kw OR (Pyemias):ab,ti,kw OR (Pyohemia):ab,ti,kw OR (Pyohemias):ab,ti,kw OR (Pyaemia):ab,ti,kw OR (Pyaemias):ab,ti,kw OR (Septicemia):ab,ti,kw OR (Septicemias):ab,ti,kw OR (Poisoning, Blood):ab,ti,kw OR (Blood Poisoning):ab,ti,kw OR (Blood Poisonings):ab,ti,kw OR (Poisonings, Blood):ab,ti,kw OR (septic shock):ab,ti,kw OR (sepsis-associated hypotension):ab,ti,kw OR (septic disease):ab,ti,kw OR (Shock, Septic):ab,ti,kw OR (septic):ab,ti,kw | 7952 |
| #3 | #1 OR #2 | 10926 |
| #4 | MeSH descriptor: [Machine Learning] explode all trees | 252 |
| #5 | (Transfer Learning):ab,ti,kw OR (Deep learning):ab,ti,kw OR (Learning, Transfer):ab,ti,kw OR (Ensemble Learning):ab,ti,kw OR (artificial intelligence):ab,ti,kw OR (Prediction model):ab,ti,kw OR (random forest):ab,ti,kw OR (artificial neural network):ab,ti,kw OR (ANN):ab,ti,kw OR (Support vector machine):ab,ti,kw OR (SVM):ab,ti,kw OR (Gradient Boosting Machine):ab,ti,kw OR (GBM):ab,ti,kw OR (Nomogram):ab,ti,kw OR (XGboost):ab,ti,kw OR (Decision tree):ab,ti,kw OR (Development and validation):ab,ti,kw OR (Risk Prediction):ab,ti,kw OR (Risk-Prediction):ab,ti,kw OR (machine intelligence):ab,ti,kw OR (bayesian):ab,ti,kw OR (naïve bayes):ab,ti,kw OR (decision support):ab,ti,kw OR (adaboost):ab,ti,kw OR (Learning, Machine):ab,ti,kw OR (learning machine):ab,ti,kw OR (learning machines):ab,ti,kw | 27486 |
| #6 | #4 OR #5 | 27486 |
| #7 | MeSH descriptor: [Mortality] explode all trees | 14058 |
| #8 | (Mortalities):ab,ti,kw OR (Case Fatality Rate):ab,ti,kw OR (Case Fatality Rates):ab,ti,kw OR (Rate, Case Fatality):ab,ti,kw OR (Rates, Case Fatality):ab,ti,kw OR (CFR Case Fatality Rate):ab,ti,kw OR (mors):ab,ti,kw OR (death):ab,ti,kw OR (survival):ab,ti,kw | 169040 |
| #9 | #7 OR #8 | 170208 |
| #10 | #3 AND #6 AND #9 | 63 |

**3.Embase**

| Search number | Query | Results |
| --- | --- | --- |
| #1 | 'sepsis'/exp | 318235 |
| #2 | sepsis:ab,ti OR 'bloodstream infection':ab,ti OR 'bloodstream infections':ab,ti OR 'infection, bloodstream':ab,ti OR pyemia:ab,ti OR pyemias:ab,ti OR pyohemia:ab,ti OR pyohemias:ab,ti OR pyaemia:ab,ti OR pyaemias:ab,ti OR septicemia:ab,ti OR septicemias:ab,ti OR 'poisoning, blood':ab,ti OR 'blood poisoning':ab,ti OR 'blood poisonings':ab,ti OR 'poisonings, blood':ab,ti OR 'septic disease':ab,ti OR 'septic shock':ab,ti OR 'sepsis-associated hypotension':ab,ti OR 'shock, septic':ab,ti OR septic:ab,ti | 257290 |
| #3 | #1 OR #2 | 399615 |
| #4 | 'machine learning'/exp | 325102 |
| #5 | 'machine learning':ab,ti OR 'transfer learning':ab,ti OR 'deep learning':ab,ti OR 'learning, transfer':ab,ti OR 'ensemble learning':ab,ti OR 'artificial intelligence':ab,ti OR 'prediction model':ab,ti OR 'random forest':ab,ti OR 'artificial neural network':ab,ti OR ann:ab,ti OR 'support vector machine':ab,ti OR svm:ab,ti OR 'gradient boosting machine':ab,ti OR gbm:ab,ti OR nomogram:ab,ti OR xgboost:ab,ti OR 'decision tree':ab,ti OR (development:ab,ti AND validation:ab,ti) OR 'risk prediction':ab,ti OR 'machine intelligence':ab,ti OR bayesian:ab,ti OR 'naïve bayes':ab,ti OR 'decision support':ab,ti OR adaboost:ab,ti OR 'learning, machine':ab,ti OR 'learning machine':ab,ti OR 'learning machines':ab,ti | 463196 |
| #6 | #4 OR #5 | 654430 |
| #7 | 'mortality'/exp | 1295816 |
| #8 | mortality:ab,ti OR mortalities:ab,ti OR 'case fatality rate':ab,ti OR 'case fatality rates':ab,ti OR 'rate, case fatality':ab,ti OR 'rates, case fatality':ab,ti OR 'cfr case fatality rate':ab,ti OR mors:ab,ti OR death:ab,ti OR survival:ab,ti | 3485679 |
| #9 | #7 OR #8 | 3800778 |
| #10 | #3 AND #6 AND #9 | 2245 |

**4.Web of science**

| Search number | Query | Results |
| --- | --- | --- |
| #1 | TS=(sepsis OR Bloodstream Infection OR Bloodstream Infections OR Infection, Bloodstream OR Pyemia OR Pyemias OR Pyohemia OR Pyohemias OR Pyaemia OR Pyaemias OR Septicemia OR Septicemias OR Poisoning, Blood OR Blood Poisoning OR Blood Poisonings OR Poisonings, Blood OR septic shock OR sepsis-associated hypotension OR septic disease OR Shock, Septic OR septic) | 218874 |
| #2 | TS=(machine learning OR Transfer Learning OR Deep learning OR Learning, Transfer OR Ensemble Learning OR artificial intelligence OR Prediction model OR random forest OR artificial neural network OR ANN OR Support vector machine OR SVM OR Gradient Boosting Machine OR GBM OR Nomogram OR XGboost OR Decision tree OR Development and validation OR Risk Prediction OR Risk-Prediction OR machine intelligence OR bayesian OR naïve bayes OR decision support OR adaboost OR Learning, Machine OR learning machine OR learning machines) | 2210995 |
| #3 | TS=(mortality OR Mortalities OR Case Fatality Rate OR Case Fatality Rates OR Rate, Case Fatality OR Rates, Case Fatality OR CFR Case Fatality Rate OR mors OR death OR survival) | 3290561 |
| #4 | #1 AND #2 AND #3 | 3390 |

**Table S2.** Overview of key characteristics per study.

| **First author** | **Year** | **Country** | **Study design** | **Source of patients** | **Diagnostic criteria for sepsis** | **Death time** | **Number of deaths** | **Total number of samples** | **Number of samples in the training cohort** | **Generation of the validation cohort** | **Number of samples in the validation cohort** | **Processing method for missing values** | **Variable selection/feature selection methods** | **Model type** |
| --- | --- | --- | --- | --- | --- | --- | --- | --- | --- | --- | --- | --- | --- | --- |
| Deyuan Zhi | 2021 | China | Case-control study | MIMIC-Ⅲ | Sepsis 3.0 | Death in hospital | 862 | 1964 | 1839 | External validation | 125 | Deletion | Univariate | LR, RF |
| Chenyan Zhao | 2020 | China | Case-control study | MIMIC-Ⅲ | Sepsis 3.0 | Death within 1 month | 1182 | 5663 | 3964 | Random sampling | 1699 |  | Univariate and multivariate logistic regression | GBM, LR |
| Luming Zhang | 2022 | China | Case-control study | MIMIC-IV |  | Death within 1 month |  | 6503 | 5202 | Random sampling | 1301 | Multiple imputation | Univariate + multivariate | RF |
| Kai Zhang | 2020 | China | Case-control study | MIMIC-Ⅲ | Sepsis 3.0 | Death within 1 month | 2042 | 11101 | 5443 | External validation | 5658 | Deletion | LASSO | LR, MARS model, RF, XGBoost |
| Zhixuan Zeng | 2021 | China | Retrospective cohort study | MIMIC-III eICU-CRD | Sepsis 3.0 | Death in hospital | 4422 | 24653 | 12558 | External validation | 12095 | KNN imputation | Stepwise logistic regression | BM_total, LR |
| Qingbo Zeng | 2021 | China | Single-center retrospective study | Single-center | Sepsis 3.0 | Death within 3 month | 72 | 231 | 161 | Random sampling | 70 |  | Multivariate logistic regression, stepwise algorithm | LR |
| Ren-qi Yao | 2020 | China | Single-center retrospective study | MIMIC-Ⅲ | Sepsis 3.0 | Death in hospital | 397 | 3713 | 3713 | 5-fold cross-validation |  | Multiple imputation | Stepwise logistic regression | XGBoost, LR |
| Hector R. Wong, MD | 2014 | America | Prospective, observational, multi-center cohort study | Multi-center | Sepsis 2.0 | Death within 1 month | 208 | 672 | 341 | External validation | 331 |  |  | DT |
| Bernhard Wernly | 2020 | Austria | Case-control study | eICU, MIMIC-III | Angus criteria | Death in hospital |  | 13634 |  | Random sampling, cross-validation | MIMIC-III: 9655  eICU: 3979 | Forward filling strategy |  | ANN, LR |
| Hanyin Wang | 2022 | USA |  | MIMIC-III v1.4 | Sepsis 3.0 | Death in hospital | 836 | 5783 | 4048 | Random sampling | 1735 | Deletion |  | Ridge regression, ANN, Passive-aggressive, kNN, RF, SVM, SGDClassifier, NB, LR |
| Bin Wang | 2021 | China | Retrospective cohort study | Single-center | Sepsis 3.0 | Death within 1 month | 212 | 1023 | 717 | External validation | 306 | Deletion | Forward stepwise method | LR |
| William P. T. M. van Doorn | 2021 | Netherlands | Single-center retrospective cohort study | Single-center | Sepsis 3.0 | Death within 1 month | 174 | 1344 | 1244 | 5-fold cross-validation | 100 | Machine learning model | Univariate + multivariate | XGBoost |
| Saraschandra Vallabhajosyula | 2018 | USA | Retrospective cohort study | Large database | Sepsis 3.0 | Death within 1 month Death within 1 year | Death within 1 month:2416 Death within 1 year:2660 | 5352 | 4033 | Random sampling | 1319 |  | Regression analysis with backward stepwise variable selection | MAVIC model |
| Athanasios Tsoukalas | 2015 | USA | Retrospective study | Large database | Sepsis 1.0 | Unclear |  | 745 | 745 | 10-fold cross-validation |  |  |  | SVM |
| R. Andrew Taylor, MD | 2016 | USA | Retrospective study | Multi-center | Sepsis 1.0 | Death in hospital | 260 | 5278 | 4222 | Random sampling | 1056 | Missiong values were processed within models | Univariate | RF, DT, LR |
| Ishan Taneja | 2021 | USA | Prospective observational cohort study | Multi-center | Sepsis 3.0 | Death within 1 month | 71 | 1400 | 933 | Random sampling | 467 | Imputation with data from the training set | Variable importance ranking | RF |
| Longxiang Su | 2021 | China | Retrospective study | Single-center | Sepsis 3.0 | Unclear | 415 | 2224 | 1557 | Random sampling | 667 | KNN(K=5) | LASSO | LR, RF, XGBoost |
| Jaime L Speiser | 2018 | Canada | A nested cohort study | CATSS Database | Sepsis 1.0 | Death in hospital | 2141 | 4222 | 2111 | Random sampling | 2111 | Surrogate splitting method |  | DT |
| Mas’uud Ibnu Samsudin, MD | 2018 | Singapore | Retrospective case-control study | Single-center | Sepsis 1.0 | Death within 1 month | 40 | 214 | 214 |  |  |  | Forward selection stepwise logistic regression | SEDS model |
| Andrés Rodríguez, MSc | 2020 | Colombia | Prospective cohort | Multi-center | Sepsis 1.0 | Death in hospital | 289 | 2510 | 2008 | Random sampling 10-fold cross-validation | 502 | Deletion |  | DT, RF, ANN , SVM |
| Yinlong Ren | 2022 | China | Retrospective case-control study | MIMIC-III | Sepsis 3.0 | Death in hospital | 485 | 1676 | 1173 | Random sampling | 503 | Multiple imputation | Stepwise logistic regression | LR |
| Sumanth Madhusudan Prabhakar | 2019 | Singapore | Retrospective case-control study | Single-center |  | Death within 1 month | 86 | 343 | 343 |  |  |  |  | LR |
| Jau-Woei Perng | 2019 | Taiwan | Retrospective case-control study | Private database | Sepsis 1.0 | Death in hospital Death within 1 month | Death in hospital:1991 Death within 1 month:5939 | 42220 | 29554 | Random sampling K-fold cross-validation | 12666 | Mean or median replacement,construction of an L1 or L2 constraint in the neural network | RF | RF, KNN, SVM, Softmax |
| James Yeongjun Park | 2022 | USA | Prospective cohort study | NIS database | Angus criteria | Death in hospital | 196,841 | 923,759 | 704246 | External validation | 219513 | Deletion | Variable importance of the random forest by Gini Impurity and Xgboost Model by SHAP | LR, LASSO, RF Xgboost, ANN, Super Learner |
| Nan Liu | 2021 | Singapore | Retrospective cohort study | Single-center | Sepsis 3.0 | Death within 1 month | 66 | 342 | 342 | 10-fold cross-validation |  | Median imputation | Backward stepwise multivariate logistic regression | HRnV model; |
| Hui Liu | 2020 | China | Retrospective cohort study | MIMIC-III | Sepsis 3.0 | Death within 1 month Death within 2-month Death within 3 month |  | 5240 | 3667 | Random sampling | 1573 | Multiple difference complement method | Backward stepwise selection method with Cox regression | Survival model |
| Ke Li, PhD | 2021 | China |  | (MIMIC-III) V1.4 | ICD-9-CM | Death in hospital | 1352 | 3937 | 3937 | 5-fold cross-validation |  | Mean imputation | DT | DT, LR, KNN, RF, SVM |
| Tara Lagu, MD, MPH | 2011 | USA |  | Multi-center | ICD-9-CM | Death in hospital | 33308 | 167288 | 166931 | External validation | 357 |  | All clinically relevant variables | LR |
| Guilan Kong | 2020 | China |  | MIMIC-III | Sepsis 3.0 | Death in hospital | 2949 | 16688 | 16688 | five-fold CV method |  | Mean imputation | Univariate | LASSO, RF, GBM, LR |
| Adam Karlsson | 2021 | Sweden | Retrospective cross-sectional design | Single-center | Sepsis 2.0 | Death in hospital Death within 1 month | Death in hospital:63 Death within 1 month: 98 | 445 | 356 | 10-fold cross-validation | 89 |  | Exclusion of the least important variable in Gini Impurity | RF |
| Fabián Jaimes | 2005 | Colombia | Longitudinal cohort study | Multi-center | Sepsis 1.0 | Death within 1 month | 101 | 533 | 400 | Random sampling | 133 | Median imputation | Univariate | LR, ANN |
| Chang Hu | 2022 |  | Retrospective modeling study | MIMIC-IV, v.1.0 | Sepsis 3.0 | Death in hospital | 1107 | 8817 | 7054 | Random sampling | 1763 | Multiple imputation | LASSO | SVM, KNN, XGBoost, DT, NB, RF, LR |
| Jen-Fu Hsu | 2021 | Taiwan | Prospective observational study | Single-center | Sepsis 1.0 | Death in hospital | 90 | 1095 | 765 | Random sampling | 330 |  |  | kNN, SVM, RF, XGB, ANN, DT, DNN |
| Nianzong Hou | 2020 | China |  | MIMIC-III v1.4 | Sepsis 3.0 | Death within 1 month | 889 | 4559 |  |  |  | Multiple imputation | Stepwise selection method | LR, XGBoost |
| Satyen Hargovan, MBBS (Hons) | 2020 | Australia | Retrospective cohort study | Cairns Hospital (single-center) | Sepsis 3.0 | Death in hospital | 59 | 500 |  | Simple sampling method |  |  | Multivariable backward stepwise logistic regression | 4-Hour Cairns Sepsis Model |
| Shadi Ghiasi | 2022 | UK | Prospective study | Single-center | Sepsis 3.0 | Death in hospital | 14 | 40 |  | Cross-validation |  |  |  | SVM, Gaussian process classification model, XGBoost, ANN |
| J.E. García-Gallo | 2018 | Colombia | Retrospective register-based cohort study | MIMIC-III | Sepsis 3.0 | Death within 1 year | 2450 | 5650 | 3955 | Random sampling | 1695 | Missing values were precossed within models | LASSO, Stochastic Gradient Boosting (SGB) | XGBoost |
| Dee W. Ford, MD, MSCR | 2016 | Carolina | Retrospective cohort study | Multi-center | ICD-9-CM | Death in hospital |  | 563155 | 65069 | Random sampling External validation | Random sampling: 43379 Martin cohort: 139049 Angus Cohort: 523637 |  | Univariate | LR |
| Mengshi Chen, MD | 2017 | China | Retrospective case-control study | Single-center | Sepsis 3.0 | Unclear | 210 | 788 | 592 | Random sampling | 197 | Deletion | Forward stepwise method | LR |
| Hsiao-Yun Chao | 2022 | Taiwan | Prospective observational study | Multi-center | Sepsis 1.0和Sepsis 3.0 | Death within 1 month | 45 | 555 | 389 | Random sampling | 166 | Replacement of missing values with medians for continuous features and modes for categorical features | RF | XGBoost, RF, SVM, ANN, DNN, LR |
| Gary S. Phillips, MAS | 2015 | USA | Retrospective observational study | Multi-center | Sepsis 2.0: The definitions of severe sepsis and septic shock are from the Second Definitions Conference | Death in hospital |  | 43203 | 38884 | Random sampling | 4319 | Single imputation |  | LR |
| Vicent J. Ribas Ripoll | 2014 | Spain |  | MIMIC II |  | Unclear | 84 | 400 |  | 10-fold cross-validation |  | No missing values |  | LR |
| Xi Guo | 2022 | China | Retrospective observational study | Single-center | Sepsis 3.0 | Death within 1 month | 49 | 141 |  |  |  |  | A combination of clinical values and potential confounders | Survival model |
| Minghui Gong | 2022 | China |  | MIMIC-IV eICU | Sepsis 3.0 | Death in hospital | 5113 | 46140 | 16520 | External validation | 29620 | Deletion |  | ANN, SVM, LR, XGBoost |
| Javier E. Garcí a-Gallo | 2019 | Colombia |  | MIMIC III | Angus criteria | Death within 1 year | 2446 | 5650 | 3955 | Random sampling | 1695 |  |  | DT |
| Xianfei Ding | 2022 | China |  | Single-center | sepsis 3.0 | Death in hospital Death within 1 month Death within 3 month | Death in hospital:53 Death within 1 month:49 Death within 3 month:54 | 96 |  |  |  |  | Univariate | model 1, model 2, model 3 |
| Wenxin Wang | 2021 | China |  | Single-center | Sepsis 3.0 the International Guidelines for Sepsis and Septic Shock: 2016 | Death within 1 month | 31 | 118 |  |  |  |  | Univariate | The combined prediction model of septic shock based on PCT, suP AR, AP ACHE II, and SOF A scores |
| Lifeng Wang | 2021 | China | Prospective single-center observational study | Single-center | Sepsis 3.0 | Death within 1 month | 41 | 175 |  |  |  |  | Univariate + multivariate | LR |
| Mehtap Selcuk | 2022 | Turkey | Retrospective study | Single-center | Sepsis 3.0 | Death in hospital | 67 | 200 |  | Random sampling K-fold cross-validation |  | No missing values |  | LR, SVM, DT, RF, XGBoost, KNN, ANN, GNB |
| Vicent J. Ribas | 2018 | Spain | Prospective observational cohort study | Large database: SODIR |  | Death in hospital | 104 | 354 |  |  |  |  |  | SVM |

**Table S3** Risk of bias assessment results

| **Author** | **Year** | **Participants** | | | | | **Predictors** | | | | | | **Outcomes** | | | | | | | | | **Analysis** | | | | | | | | | | | |
| --- | --- | --- | --- | --- | --- | --- | --- | --- | --- | --- | --- | --- | --- | --- | --- | --- | --- | --- | --- | --- | --- | --- | --- | --- | --- | --- | --- | --- | --- | --- | --- | --- | --- |
|  |  | **question1** | **question2** | **High ROB** | **Low ROB** | **ALL** | **question1** | **question2** | **question3** | **High ROB** | **Low ROB** | **ALL** | **question1** | **question2** | **question3** | **question4** | **question5** | **question6** | **High ROB** | **Low ROB** | **ALL** | **question1** | **question2** | **question3** | **question4** | **question5** | **question6** | **question7** | **question8** | **question9** | **High ROB** | **Low ROB** | **ALL** |
| Deyuan Zhi | 2021 | 1 | 1 | 0 | 1 | 1 | 1 | 1 | 1 | 0 | 1 | 1 | 1 | 1 | 1 | 1 | 1 | 1 | 0 | 1 | 1 | 1 | 1 | 1 | 2 | 2 | 0 | 1 | 0 | 1 | 2 | 1 | 2 |
|  |  | 1 | 1 | 0 | 1 | 1 | 1 | 1 | 1 | 0 | 1 | 1 | 1 | 1 | 1 | 1 | 1 | 1 | 0 | 1 | 1 | 1 | 1 | 1 | 2 | 2 | 0 | 1 | 0 | 1 | 2 | 1 | 2 |
| Chenyan Zhao | 2020 | 1 | 1 | 0 | 1 | 1 | 1 | 2 | 0 | 2 | 1 | 2 | 1 | 1 | 1 | 1 | 1 | 1 | 0 | 1 | 1 | 1 | 1 | 1 | 2 | 1 | 0 | 1 | 0 | 1 | 2 | 0 | 2 |
|  |  | 1 | 1 | 0 | 1 | 1 | 1 | 2 | 0 | 2 | 1 | 2 | 1 | 1 | 1 | 1 | 1 | 1 | 0 | 1 | 1 | 1 | 1 | 1 | 2 | 1 | 0 | 1 | 0 | 1 | 2 | 0 | 2 |
| Luming Zhang | 2022 | 1 | 1 | 0 | 1 | 1 | 1 | 1 | 1 | 0 | 1 | 1 | 1 | 1 | 1 | 1 | 1 | 1 | 0 | 1 | 1 | 0 | 1 | 1 | 1 | 1 | 0 | 1 | 0 | 1 | 0 | 0 | 0 |
| Kai Zhang | 2020 | 1 | 1 | 0 | 1 | 1 | 1 | 1 | 1 | 0 | 1 | 1 | 1 | 1 | 1 | 1 | 1 | 1 | 0 | 1 | 1 | 1 | 1 | 1 | 2 | 1 | 0 | 1 | 0 | 1 | 2 | 0 | 2 |
|  |  | 1 | 1 | 0 | 1 | 1 | 1 | 1 | 1 | 0 | 1 | 1 | 1 | 1 | 1 | 1 | 1 | 1 | 0 | 1 | 1 | 1 | 1 | 1 | 2 | 1 | 0 | 1 | 0 | 1 | 2 | 0 | 2 |
|  |  | 1 | 1 | 0 | 1 | 1 | 1 | 1 | 1 | 0 | 1 | 1 | 1 | 1 | 1 | 1 | 1 | 1 | 0 | 1 | 1 | 1 | 1 | 1 | 2 | 1 | 0 | 1 | 0 | 1 | 2 | 0 | 2 |
|  |  | 1 | 1 | 0 | 1 | 1 | 1 | 1 | 1 | 0 | 1 | 1 | 1 | 1 | 1 | 1 | 1 | 1 | 0 | 1 | 1 | 1 | 1 | 1 | 2 | 1 | 0 | 1 | 0 | 1 | 2 | 0 | 2 |
| Zhixuan Zeng | 2021 | 1 | 1 | 0 | 1 | 1 | 1 | 1 | 1 | 0 | 1 | 1 | 1 | 1 | 1 | 1 | 1 | 1 | 0 | 1 | 1 | 1 | 1 | 1 | 1 | 1 | 0 | 1 | 0 | 1 | 0 | 0 | 0 |
|  |  | 1 | 1 | 0 | 1 | 1 | 1 | 1 | 1 | 0 | 1 | 1 | 1 | 1 | 1 | 1 | 1 | 1 | 0 | 1 | 1 | 1 | 1 | 1 | 1 | 1 | 0 | 1 | 0 | 1 | 0 | 0 | 0 |
|  |  | 1 | 1 | 0 | 1 | 1 | 1 | 1 | 1 | 0 | 1 | 1 | 1 | 1 | 1 | 1 | 1 | 1 | 0 | 1 | 1 | 1 | 1 | 1 | 1 | 1 | 0 | 1 | 0 | 1 | 0 | 0 | 0 |
|  |  | 1 | 1 | 0 | 1 | 1 | 1 | 1 | 1 | 0 | 1 | 1 | 1 | 1 | 1 | 1 | 1 | 1 | 0 | 1 | 1 | 1 | 1 | 1 | 1 | 1 | 0 | 1 | 0 | 1 | 0 | 0 | 0 |
| Qingbo Zeng | 2021 | 2 | 1 | 2 | 0 | 2 | 1 | 0 | 1 | 0 | 0 | 0 | 1 | 1 | 1 | 1 | 1 | 1 | 0 | 1 | 1 | 2 | 1 | 1 | 2 | 1 | 0 | 1 | 0 | 1 | 2 | 1 | 2 |
| Ren-qi Yao | 2020 | 1 | 1 | 0 | 1 | 1 | 1 | 1 | 1 | 0 | 1 | 1 | 1 | 1 | 1 | 1 | 1 | 0 | 0 | 0 | 0 | 2 | 1 | 1 | 1 | 1 | 1 | 1 | 0 | 1 | 2 | 1 | 2 |
|  |  | 1 | 1 | 0 | 1 | 1 | 1 | 1 | 1 | 0 | 1 | 1 | 1 | 1 | 1 | 1 | 1 | 0 | 0 | 0 | 0 | 2 | 1 | 1 | 1 | 1 | 1 | 1 | 0 | 1 | 2 | 1 | 2 |
| Hector R. Wong, MD | 2014 | 1 | 1 | 0 | 1 | 1 | 1 | 1 | 1 | 0 | 1 | 1 | 1 | 1 | 1 | 1 | 1 | 1 | 0 | 1 | 1 | 1 | 1 | 1 | 1 | 1 | 0 | 1 | 0 | 1 | 0 | 0 | 0 |
| Hanyin Wang | 2022 | 1 | 1 | 0 | 1 | 1 | 1 | 1 | 1 | 0 | 1 | 1 | 1 | 1 | 1 | 1 | 1 | 1 | 0 | 1 | 1 | 1 | 1 | 1 | 2 | 1 | 0 | 1 | 1 | 1 | 2 | 1 | 2 |
|  |  | 1 | 1 | 0 | 1 | 1 | 1 | 1 | 1 | 0 | 1 | 1 | 1 | 1 | 1 | 1 | 1 | 1 | 0 | 1 | 1 | 1 | 1 | 1 | 2 | 1 | 0 | 1 | 1 | 1 | 2 | 1 | 2 |
|  |  | 1 | 1 | 0 | 1 | 1 | 1 | 1 | 1 | 0 | 1 | 1 | 1 | 1 | 1 | 1 | 1 | 1 | 0 | 1 | 1 | 1 | 1 | 1 | 2 | 1 | 0 | 1 | 1 | 1 | 2 | 1 | 2 |
|  |  | 1 | 1 | 0 | 1 | 1 | 1 | 1 | 1 | 0 | 1 | 1 | 1 | 1 | 1 | 1 | 1 | 1 | 0 | 1 | 1 | 1 | 1 | 1 | 2 | 1 | 0 | 1 | 1 | 1 | 2 | 1 | 2 |
|  |  | 1 | 1 | 0 | 1 | 1 | 1 | 1 | 1 | 0 | 1 | 1 | 1 | 1 | 1 | 1 | 1 | 1 | 0 | 1 | 1 | 1 | 1 | 1 | 2 | 1 | 0 | 1 | 1 | 1 | 2 | 1 | 2 |
|  |  | 1 | 1 | 0 | 1 | 1 | 1 | 1 | 1 | 0 | 1 | 1 | 1 | 1 | 1 | 1 | 1 | 1 | 0 | 1 | 1 | 1 | 1 | 1 | 2 | 1 | 0 | 1 | 1 | 1 | 2 | 1 | 2 |
|  |  | 1 | 1 | 0 | 1 | 1 | 1 | 1 | 1 | 0 | 1 | 1 | 1 | 1 | 1 | 1 | 1 | 1 | 0 | 1 | 1 | 1 | 1 | 1 | 2 | 1 | 0 | 1 | 1 | 1 | 2 | 1 | 2 |
|  |  | 1 | 1 | 0 | 1 | 1 | 1 | 1 | 1 | 0 | 1 | 1 | 1 | 1 | 1 | 1 | 1 | 1 | 0 | 1 | 1 | 1 | 1 | 1 | 2 | 1 | 0 | 1 | 1 | 1 | 2 | 1 | 2 |
|  |  | 1 | 1 | 0 | 1 | 1 | 1 | 1 | 1 | 0 | 1 | 1 | 1 | 1 | 1 | 1 | 1 | 1 | 0 | 1 | 1 | 1 | 1 | 1 | 2 | 1 | 0 | 1 | 1 | 1 | 2 | 1 | 2 |
|  |  | 1 | 1 | 0 | 1 | 1 | 1 | 1 | 1 | 0 | 1 | 1 | 1 | 1 | 1 | 1 | 1 | 1 | 0 | 1 | 1 | 1 | 1 | 1 | 2 | 1 | 0 | 1 | 1 | 1 | 2 | 1 | 2 |
|  |  | 1 | 1 | 0 | 1 | 1 | 1 | 1 | 1 | 0 | 1 | 1 | 1 | 1 | 1 | 1 | 1 | 1 | 0 | 1 | 1 | 1 | 1 | 1 | 2 | 1 | 0 | 1 | 1 | 1 | 2 | 1 | 2 |
|  |  | 1 | 1 | 0 | 1 | 1 | 1 | 1 | 1 | 0 | 1 | 1 | 1 | 1 | 1 | 1 | 1 | 1 | 0 | 1 | 1 | 1 | 1 | 1 | 2 | 1 | 0 | 1 | 1 | 1 | 2 | 1 | 2 |
|  |  | 1 | 1 | 0 | 1 | 1 | 1 | 1 | 1 | 0 | 1 | 1 | 1 | 1 | 1 | 1 | 1 | 1 | 0 | 1 | 1 | 1 | 1 | 1 | 2 | 1 | 0 | 1 | 1 | 1 | 2 | 1 | 2 |
|  |  | 1 | 1 | 0 | 1 | 1 | 1 | 1 | 1 | 0 | 1 | 1 | 1 | 1 | 1 | 1 | 1 | 1 | 0 | 1 | 1 | 1 | 1 | 1 | 2 | 1 | 0 | 1 | 1 | 1 | 2 | 1 | 2 |
|  |  | 1 | 1 | 0 | 1 | 1 | 1 | 1 | 1 | 0 | 1 | 1 | 1 | 1 | 1 | 1 | 1 | 1 | 0 | 1 | 1 | 1 | 1 | 1 | 2 | 1 | 0 | 1 | 1 | 1 | 2 | 1 | 2 |
|  |  | 1 | 1 | 0 | 1 | 1 | 1 | 1 | 1 | 0 | 1 | 1 | 1 | 1 | 1 | 1 | 1 | 1 | 0 | 1 | 1 | 1 | 1 | 1 | 2 | 1 | 0 | 1 | 1 | 1 | 2 | 1 | 2 |
| Bin Wang | 2021 | 2 | 1 | 2 | 0 | 2 | 1 | 0 | 1 | 0 | 0 | 0 | 1 | 1 | 1 | 1 | 1 | 1 | 0 | 1 | 1 | 1 | 1 | 1 | 2 | 1 | 0 | 1 | 0 | 1 | 2 | 0 | 2 |
| William P. T. M. van Doorn | 2021 | 2 | 1 | 2 | 0 | 2 | 1 | 0 | 1 | 0 | 0 | 0 | 1 | 1 | 1 | 1 | 1 | 1 | 0 | 1 | 1 | 1 | 1 | 1 | 1 | 1 | 0 | 1 | 0 | 1 | 0 | 0 | 0 |
|  |  | 2 | 1 | 2 | 0 | 2 | 1 | 0 | 1 | 0 | 0 | 0 | 1 | 1 | 1 | 1 | 1 | 1 | 0 | 1 | 1 | 1 | 1 | 1 | 1 | 1 | 0 | 1 | 0 | 1 | 0 | 0 | 0 |
| Saraschandra Vallabhajosyula | 2018 | 2 | 1 | 2 | 0 | 2 | 1 | 0 | 1 | 0 | 0 | 0 | 1 | 1 | 1 | 1 | 1 | 1 | 0 | 1 | 1 | 1 | 1 | 1 | 2 | 1 | 0 | 1 | 0 | 1 | 2 | 0 | 2 |
| Athanasios Tsoukalas | 2015 | 2 | 1 | 2 | 0 | 2 | 1 | 0 | 1 | 0 | 0 | 0 | 1 | 1 | 1 | 1 | 1 | 1 | 0 | 1 | 1 | 2 | 1 | 1 | 2 | 1 | 0 | 1 | 0 | 1 | 2 | 1 | 2 |
| R. Andrew Taylor, MD | 2016 | 2 | 1 | 2 | 0 | 2 | 1 | 0 | 1 | 0 | 0 | 0 | 1 | 1 | 1 | 1 | 1 | 0 | 0 | 0 | 0 | 1 | 1 | 1 | 1 | 2 | 0 | 1 | 1 | 1 | 2 | 1 | 2 |
|  |  | 2 | 1 | 2 | 0 | 2 | 1 | 0 | 1 | 0 | 0 | 0 | 1 | 1 | 1 | 1 | 1 | 0 | 0 | 0 | 0 | 1 | 1 | 1 | 1 | 2 | 0 | 1 | 1 | 1 | 2 | 1 | 2 |
|  |  | 2 | 1 | 2 | 0 | 2 | 1 | 0 | 1 | 0 | 0 | 0 | 1 | 1 | 1 | 1 | 1 | 0 | 0 | 0 | 0 | 1 | 1 | 1 | 1 | 2 | 0 | 1 | 1 | 1 | 2 | 1 | 2 |
| Ishan Taneja | 2021 | 1 | 1 | 0 | 1 | 1 | 1 | 1 | 1 | 0 | 1 | 1 | 1 | 1 | 1 | 1 | 1 | 1 | 0 | 1 | 1 | 1 | 1 | 1 | 2 | 1 | 0 | 1 | 1 | 1 | 2 | 1 | 2 |
| Longxiang Su | 2021 | 2 | 1 | 2 | 0 | 2 | 1 | 0 | 1 | 0 | 0 | 0 | 1 | 1 | 1 | 1 | 1 | 0 | 0 | 0 | 0 | 1 | 1 | 1 | 1 | 1 | 0 | 1 | 1 | 1 | 0 | 0 | 0 |
|  |  | 2 | 1 | 2 | 0 | 2 | 1 | 0 | 1 | 0 | 0 | 0 | 1 | 1 | 1 | 1 | 1 | 0 | 0 | 0 | 0 | 1 | 1 | 1 | 1 | 1 | 0 | 1 | 1 | 1 | 0 | 0 | 0 |
|  |  | 2 | 1 | 2 | 0 | 2 | 1 | 0 | 1 | 0 | 0 | 0 | 1 | 1 | 1 | 1 | 1 | 0 | 0 | 0 | 0 | 1 | 1 | 1 | 1 | 1 | 0 | 1 | 1 | 1 | 0 | 0 | 0 |
| Jaime L Speiser | 2018 | 1 | 1 | 0 | 1 | 1 | 1 | 0 | 1 | 0 | 0 | 0 | 1 | 1 | 1 | 1 | 1 | 1 | 0 | 1 | 1 | 1 | 1 | 1 | 1 | 0 | 0 | 1 | 1 | 1 | 0 | 0 | 0 |
| Mas’uud Ibnu Samsudin, MD | 2018 | 2 | 1 | 2 | 0 | 2 | 1 | 0 | 1 | 0 | 0 | 0 | 1 | 1 | 1 | 1 | 1 | 1 | 0 | 1 | 1 | 2 | 1 | 1 | 1 | 1 | 0 | 1 | 0 | 1 | 2 | 0 | 2 |
| Andrés Rodríguez, MSc | 2020 | 1 | 1 | 0 | 1 | 1 | 1 | 0 | 1 | 0 | 0 | 0 | 1 | 1 | 1 | 1 | 1 | 1 | 0 | 1 | 1 | 1 | 1 | 1 | 2 | 1 | 0 | 1 | 1 | 1 | 2 | 1 | 2 |
|  |  | 1 | 1 | 0 | 1 | 1 | 1 | 0 | 1 | 0 | 0 | 0 | 1 | 1 | 1 | 1 | 1 | 1 | 0 | 1 | 1 | 1 | 1 | 1 | 2 | 1 | 0 | 1 | 1 | 1 | 2 | 1 | 2 |
|  |  | 1 | 1 | 0 | 1 | 1 | 1 | 0 | 1 | 0 | 0 | 0 | 1 | 1 | 1 | 1 | 1 | 1 | 0 | 1 | 1 | 1 | 1 | 1 | 2 | 1 | 0 | 1 | 1 | 1 | 2 | 1 | 2 |
|  |  | 1 | 1 | 0 | 1 | 1 | 1 | 0 | 1 | 0 | 0 | 0 | 1 | 1 | 1 | 1 | 1 | 1 | 0 | 1 | 1 | 1 | 1 | 1 | 2 | 1 | 0 | 1 | 1 | 1 | 2 | 1 | 2 |
|  |  | 1 | 1 | 0 | 1 | 1 | 1 | 0 | 1 | 0 | 0 | 0 | 1 | 1 | 1 | 1 | 1 | 1 | 0 | 1 | 1 | 1 | 1 | 1 | 2 | 1 | 0 | 1 | 1 | 1 | 2 | 1 | 2 |
|  |  | 1 | 1 | 0 | 1 | 1 | 1 | 0 | 1 | 0 | 0 | 0 | 1 | 1 | 1 | 1 | 1 | 1 | 0 | 1 | 1 | 1 | 1 | 1 | 2 | 1 | 0 | 1 | 1 | 1 | 2 | 1 | 2 |
|  |  | 1 | 1 | 0 | 1 | 1 | 1 | 0 | 1 | 0 | 0 | 0 | 1 | 1 | 1 | 1 | 1 | 1 | 0 | 1 | 1 | 1 | 1 | 1 | 2 | 1 | 0 | 1 | 1 | 1 | 2 | 1 | 2 |
|  |  | 1 | 1 | 0 | 1 | 1 | 1 | 0 | 1 | 0 | 0 | 0 | 1 | 1 | 1 | 1 | 1 | 1 | 0 | 1 | 1 | 1 | 1 | 1 | 2 | 1 | 0 | 1 | 1 | 1 | 2 | 1 | 2 |
|  |  | 1 | 1 | 0 | 1 | 1 | 1 | 0 | 1 | 0 | 0 | 0 | 1 | 1 | 1 | 1 | 1 | 1 | 0 | 1 | 1 | 1 | 1 | 1 | 2 | 1 | 0 | 1 | 1 | 1 | 2 | 1 | 2 |
|  |  | 1 | 1 | 0 | 1 | 1 | 1 | 0 | 1 | 0 | 0 | 0 | 1 | 1 | 1 | 1 | 1 | 1 | 0 | 1 | 1 | 1 | 1 | 1 | 2 | 1 | 0 | 1 | 1 | 1 | 2 | 1 | 2 |
| Yinlong Ren | 2022 | 1 | 1 | 0 | 1 | 1 | 1 | 1 | 1 | 0 | 1 | 1 | 1 | 1 | 1 | 1 | 1 | 0 | 0 | 0 | 0 | 1 | 1 | 1 | 2 | 1 | 0 | 1 | 0 | 1 | 2 | 0 | 2 |
| Sumanth Madhusudan Prabhakar | 2019 | 2 | 1 | 2 | 0 | 2 | 1 | 1 | 1 | 0 | 1 | 1 | 1 | 1 | 1 | 1 | 1 | 1 | 0 | 1 | 1 | 2 | 1 | 1 | 2 | 0 | 0 | 1 | 0 | 1 | 2 | 0 | 2 |
| Jau-Woei Perng | 2019 | 2 | 1 | 2 | 0 | 2 | 1 | 0 | 1 | 0 | 0 | 0 | 1 | 1 | 1 | 1 | 1 | 1 | 0 | 1 | 1 | 1 | 1 | 1 | 2 | 1 | 0 | 1 | 1 | 1 | 2 | 1 | 2 |
|  |  | 2 | 1 | 2 | 0 | 2 | 1 | 0 | 1 | 0 | 0 | 0 | 1 | 1 | 1 | 1 | 1 | 1 | 0 | 1 | 1 | 1 | 1 | 1 | 2 | 1 | 0 | 1 | 1 | 1 | 2 | 1 | 2 |
|  |  | 2 | 1 | 2 | 0 | 2 | 1 | 0 | 1 | 0 | 0 | 0 | 1 | 1 | 1 | 1 | 1 | 1 | 0 | 1 | 1 | 1 | 1 | 1 | 2 | 1 | 0 | 1 | 1 | 1 | 2 | 1 | 2 |
|  |  | 2 | 1 | 2 | 0 | 2 | 1 | 0 | 1 | 0 | 0 | 0 | 1 | 1 | 1 | 1 | 1 | 1 | 0 | 1 | 1 | 1 | 1 | 1 | 2 | 1 | 0 | 1 | 1 | 1 | 2 | 1 | 2 |
|  |  | 2 | 1 | 2 | 0 | 2 | 1 | 0 | 1 | 0 | 0 | 0 | 1 | 1 | 1 | 1 | 1 | 1 | 0 | 1 | 1 | 1 | 1 | 1 | 2 | 1 | 0 | 1 | 1 | 1 | 2 | 1 | 2 |
|  |  | 2 | 1 | 2 | 0 | 2 | 1 | 0 | 1 | 0 | 0 | 0 | 1 | 1 | 1 | 1 | 1 | 1 | 0 | 1 | 1 | 1 | 1 | 1 | 2 | 1 | 0 | 1 | 1 | 1 | 2 | 1 | 2 |
|  |  | 2 | 1 | 2 | 0 | 2 | 1 | 0 | 1 | 0 | 0 | 0 | 1 | 1 | 1 | 1 | 1 | 1 | 0 | 1 | 1 | 1 | 1 | 1 | 2 | 1 | 0 | 1 | 1 | 1 | 2 | 1 | 2 |
|  |  | 2 | 1 | 2 | 0 | 2 | 1 | 0 | 1 | 0 | 0 | 0 | 1 | 1 | 1 | 1 | 1 | 1 | 0 | 1 | 1 | 1 | 1 | 1 | 2 | 1 | 0 | 1 | 1 | 1 | 2 | 1 | 2 |
| James Yeongjun Park | 2022 | 1 | 1 | 0 | 1 | 1 | 1 | 1 | 1 | 0 | 1 | 1 | 1 | 1 | 1 | 1 | 1 | 1 | 0 | 1 | 1 | 1 | 1 | 1 | 2 | 1 | 0 | 1 | 0 | 1 | 2 | 0 | 2 |
|  |  | 1 | 1 | 0 | 1 | 1 | 1 | 1 | 1 | 0 | 1 | 1 | 1 | 1 | 1 | 1 | 1 | 1 | 0 | 1 | 1 | 1 | 1 | 1 | 2 | 1 | 0 | 1 | 0 | 1 | 2 | 0 | 2 |
|  |  | 1 | 1 | 0 | 1 | 1 | 1 | 1 | 1 | 0 | 1 | 1 | 1 | 1 | 1 | 1 | 1 | 1 | 0 | 1 | 1 | 1 | 1 | 1 | 2 | 1 | 0 | 1 | 0 | 1 | 2 | 0 | 2 |
|  |  | 1 | 1 | 0 | 1 | 1 | 1 | 1 | 1 | 0 | 1 | 1 | 1 | 1 | 1 | 1 | 1 | 1 | 0 | 1 | 1 | 1 | 1 | 1 | 2 | 1 | 0 | 1 | 0 | 1 | 2 | 0 | 2 |
|  |  | 1 | 1 | 0 | 1 | 1 | 1 | 1 | 1 | 0 | 1 | 1 | 1 | 1 | 1 | 1 | 1 | 1 | 0 | 1 | 1 | 1 | 1 | 1 | 2 | 1 | 0 | 1 | 0 | 1 | 2 | 0 | 2 |
|  |  | 1 | 1 | 0 | 1 | 1 | 1 | 1 | 1 | 0 | 1 | 1 | 1 | 1 | 1 | 1 | 1 | 1 | 0 | 1 | 1 | 1 | 1 | 1 | 2 | 1 | 0 | 1 | 0 | 1 | 2 | 0 | 2 |
| Nan Liu | 2021 | 2 | 1 | 2 | 0 | 2 | 1 | 0 | 1 | 0 | 0 | 0 | 1 | 1 | 1 | 1 | 1 | 1 | 0 | 1 | 1 | 2 | 1 | 1 | 2 | 1 | 0 | 1 | 1 | 1 | 2 | 0 | 2 |
| Hui Liu | 2020 | 1 | 1 | 0 | 1 | 1 | 1 | 1 | 1 | 0 | 1 | 1 | 1 | 1 | 1 | 1 | 1 | 1 | 0 | 1 | 1 | 2 | 1 | 1 | 1 | 1 | 0 | 1 | 0 | 1 | 2 | 0 | 2 |
| Ke Li, PhD | 2021 | 1 | 1 | 0 | 1 | 1 | 1 | 1 | 2 | 2 | 0 | 2 | 1 | 1 | 1 | 1 | 1 | 1 | 0 | 1 | 1 | 2 | 1 | 1 | 2 | 1 | 0 | 1 | 0 | 1 | 2 | 1 | 2 |
|  |  | 1 | 1 | 0 | 1 | 1 | 1 | 1 | 2 | 2 | 0 | 2 | 1 | 1 | 1 | 1 | 1 | 1 | 0 | 1 | 1 | 2 | 1 | 1 | 2 | 1 | 0 | 1 | 0 | 1 | 2 | 1 | 2 |
|  |  | 1 | 1 | 0 | 1 | 1 | 1 | 1 | 2 | 2 | 0 | 2 | 1 | 1 | 1 | 1 | 1 | 1 | 0 | 1 | 1 | 2 | 1 | 1 | 2 | 1 | 0 | 1 | 0 | 1 | 2 | 1 | 2 |
|  |  | 1 | 1 | 0 | 1 | 1 | 1 | 1 | 2 | 2 | 0 | 2 | 1 | 1 | 1 | 1 | 1 | 1 | 0 | 1 | 1 | 2 | 1 | 1 | 2 | 1 | 0 | 1 | 0 | 1 | 2 | 1 | 2 |
|  |  | 1 | 1 | 0 | 1 | 1 | 1 | 1 | 2 | 2 | 0 | 2 | 1 | 1 | 1 | 1 | 1 | 1 | 0 | 1 | 1 | 2 | 1 | 1 | 2 | 1 | 0 | 1 | 0 | 1 | 2 | 1 | 2 |
| Tara Lagu, MD, MPH | 2011 | 2 | 1 | 2 | 0 | 2 | 1 | 0 | 1 | 0 | 0 | 0 | 1 | 1 | 1 | 1 | 1 | 1 | 0 | 1 | 1 | 1 | 1 | 1 | 2 | 2 | 0 | 1 | 0 | 1 | 2 | 1 | 2 |
| Guilan Kong | 2020 | 1 | 1 | 0 | 1 | 1 | 1 | 1 | 1 | 0 | 1 | 1 | 1 | 1 | 1 | 1 | 1 | 1 | 0 | 1 | 1 | 2 | 1 | 1 | 2 | 2 | 0 | 1 | 0 | 1 | 2 | 0 | 2 |
|  |  | 1 | 1 | 0 | 1 | 1 | 1 | 1 | 1 | 0 | 1 | 1 | 1 | 1 | 1 | 1 | 1 | 1 | 0 | 1 | 1 | 2 | 1 | 1 | 2 | 2 | 0 | 1 | 0 | 1 | 2 | 0 | 2 |
|  |  | 1 | 1 | 0 | 1 | 1 | 1 | 1 | 1 | 0 | 1 | 1 | 1 | 1 | 1 | 1 | 1 | 1 | 0 | 1 | 1 | 2 | 1 | 1 | 2 | 2 | 0 | 1 | 0 | 1 | 2 | 0 | 2 |
|  |  | 1 | 1 | 0 | 1 | 1 | 1 | 1 | 1 | 0 | 1 | 1 | 1 | 1 | 1 | 1 | 1 | 1 | 0 | 1 | 1 | 2 | 1 | 1 | 2 | 2 | 0 | 1 | 0 | 1 | 2 | 0 | 2 |
| Adam Karlsson | 2021 | 2 | 1 | 2 | 0 | 2 | 1 | 0 | 1 | 0 | 0 | 0 | 1 | 1 | 1 | 1 | 1 | 1 | 0 | 1 | 1 | 2 | 1 | 1 | 2 | 1 | 0 | 1 | 1 | 1 | 2 | 0 | 2 |
| Fabián Jaimes | 2005 | 1 | 1 | 0 | 1 | 1 | 1 | 1 | 1 | 0 | 1 | 1 | 1 | 1 | 1 | 1 | 1 | 1 | 0 | 1 | 1 | 1 | 1 | 1 | 2 | 2 | 0 | 1 | 0 | 1 | 2 | 1 | 2 |
| Fabián Jaimes | 2005 | 1 | 1 | 0 | 1 | 1 | 1 | 1 | 1 | 0 | 1 | 1 | 1 | 1 | 1 | 1 | 1 | 1 | 0 | 1 | 1 | 1 | 1 | 1 | 2 | 2 | 0 | 1 | 0 | 1 | 2 | 1 | 2 |
| Chang Hu | 2022 | 1 | 1 | 0 | 1 | 1 | 1 | 1 | 1 | 0 | 1 | 1 | 1 | 1 | 1 | 1 | 1 | 1 | 0 | 1 | 1 | 1 | 1 | 1 | 1 | 1 | 0 | 1 | 1 | 1 | 0 | 0 | 0 |
|  |  | 1 | 1 | 0 | 1 | 1 | 1 | 1 | 1 | 0 | 1 | 1 | 1 | 1 | 1 | 1 | 1 | 1 | 0 | 1 | 1 | 1 | 1 | 1 | 1 | 1 | 0 | 1 | 1 | 1 | 0 | 0 | 0 |
|  |  | 1 | 1 | 0 | 1 | 1 | 1 | 1 | 1 | 0 | 1 | 1 | 1 | 1 | 1 | 1 | 1 | 1 | 0 | 1 | 1 | 1 | 1 | 1 | 1 | 1 | 0 | 1 | 1 | 1 | 0 | 0 | 0 |
|  |  | 1 | 1 | 0 | 1 | 1 | 1 | 1 | 1 | 0 | 1 | 1 | 1 | 1 | 1 | 1 | 1 | 1 | 0 | 1 | 1 | 1 | 1 | 1 | 1 | 1 | 0 | 1 | 1 | 1 | 0 | 0 | 0 |
|  |  | 1 | 1 | 0 | 1 | 1 | 1 | 1 | 1 | 0 | 1 | 1 | 1 | 1 | 1 | 1 | 1 | 1 | 0 | 1 | 1 | 1 | 1 | 1 | 1 | 1 | 0 | 1 | 1 | 1 | 0 | 0 | 0 |
|  |  | 1 | 1 | 0 | 1 | 1 | 1 | 1 | 1 | 0 | 1 | 1 | 1 | 1 | 1 | 1 | 1 | 1 | 0 | 1 | 1 | 1 | 1 | 1 | 1 | 1 | 0 | 1 | 1 | 1 | 0 | 0 | 0 |
|  |  | 1 | 1 | 0 | 1 | 1 | 1 | 1 | 1 | 0 | 1 | 1 | 1 | 1 | 1 | 1 | 1 | 1 | 0 | 1 | 1 | 1 | 1 | 1 | 1 | 1 | 0 | 1 | 1 | 1 | 0 | 0 | 0 |
| Jen-Fu Hsu | 2021 | 1 | 1 | 0 | 1 | 1 | 1 | 1 | 0 | 0 | 0 | 0 | 1 | 1 | 1 | 1 | 1 | 1 | 0 | 1 | 1 | 2 | 1 | 1 | 2 | 0 | 0 | 1 | 0 | 1 | 2 | 0 | 2 |
|  |  | 1 | 1 | 0 | 1 | 1 | 1 | 1 | 0 | 0 | 0 | 0 | 1 | 1 | 1 | 1 | 1 | 1 | 0 | 1 | 1 | 2 | 1 | 1 | 2 | 0 | 0 | 1 | 0 | 1 | 2 | 0 | 2 |
|  |  | 1 | 1 | 0 | 1 | 1 | 1 | 1 | 0 | 0 | 0 | 0 | 1 | 1 | 1 | 1 | 1 | 1 | 0 | 1 | 1 | 2 | 1 | 1 | 2 | 0 | 0 | 1 | 0 | 1 | 2 | 0 | 2 |
|  |  | 1 | 1 | 0 | 1 | 1 | 1 | 1 | 0 | 0 | 0 | 0 | 1 | 1 | 1 | 1 | 1 | 1 | 0 | 1 | 1 | 2 | 1 | 1 | 2 | 0 | 0 | 1 | 0 | 1 | 2 | 0 | 2 |
|  |  | 1 | 1 | 0 | 1 | 1 | 1 | 1 | 0 | 0 | 0 | 0 | 1 | 1 | 1 | 1 | 1 | 1 | 0 | 1 | 1 | 2 | 1 | 1 | 2 | 0 | 0 | 1 | 0 | 1 | 2 | 0 | 2 |
|  |  | 1 | 1 | 0 | 1 | 1 | 1 | 1 | 0 | 0 | 0 | 0 | 1 | 1 | 1 | 1 | 1 | 1 | 0 | 1 | 1 | 2 | 1 | 1 | 2 | 0 | 0 | 1 | 0 | 1 | 2 | 0 | 2 |
|  |  | 1 | 1 | 0 | 1 | 1 | 1 | 1 | 0 | 0 | 0 | 0 | 1 | 1 | 1 | 1 | 1 | 1 | 0 | 1 | 1 | 2 | 1 | 1 | 2 | 0 | 0 | 1 | 0 | 1 | 2 | 0 | 2 |
| Nianzong Hou | 2020 | 1 | 1 | 0 | 1 | 1 | 1 | 1 | 2 | 2 | 0 | 2 | 1 | 1 | 1 | 1 | 1 | 1 | 0 | 1 | 1 | 2 | 1 | 1 | 1 | 1 | 0 | 1 | 0 | 1 | 2 | 0 | 2 |
|  |  | 1 | 1 | 0 | 1 | 1 | 1 | 1 | 2 | 2 | 0 | 2 | 1 | 1 | 1 | 1 | 1 | 1 | 0 | 1 | 1 | 2 | 1 | 1 | 1 | 1 | 0 | 1 | 0 | 1 | 2 | 0 | 2 |
|  |  | 1 | 1 | 0 | 1 | 1 | 1 | 1 | 2 | 2 | 0 | 2 | 1 | 1 | 1 | 1 | 1 | 1 | 0 | 1 | 1 | 2 | 1 | 1 | 1 | 1 | 0 | 1 | 0 | 1 | 2 | 0 | 2 |
| Satyen Hargovan, MBBS (Hons) | 2020 | 2 | 1 | 2 | 0 | 2 | 1 | 0 | 1 | 0 | 0 | 0 | 1 | 1 | 1 | 1 | 1 | 0 | 0 | 0 | 0 | 2 | 1 | 1 | 2 | 1 | 0 | 1 | 0 | 1 | 2 | 1 | 2 |
| Shadi Ghiasi | 2022 | 1 | 1 | 0 | 1 | 1 | 1 | 1 | 0 | 0 | 0 | 0 | 1 | 1 | 1 | 1 | 1 | 1 | 0 | 1 | 1 | 2 | 1 | 1 | 2 | 0 | 0 | 1 | 0 | 1 | 2 | 0 | 2 |
|  |  | 1 | 1 | 0 | 1 | 1 | 1 | 1 | 0 | 0 | 0 | 0 | 1 | 1 | 1 | 1 | 1 | 1 | 0 | 1 | 1 | 2 | 1 | 1 | 2 | 0 | 0 | 1 | 0 | 1 | 2 | 0 | 2 |
|  |  | 1 | 1 | 0 | 1 | 1 | 1 | 1 | 0 | 0 | 0 | 0 | 1 | 1 | 1 | 1 | 1 | 1 | 0 | 1 | 1 | 2 | 1 | 1 | 2 | 0 | 0 | 1 | 0 | 1 | 2 | 0 | 2 |
|  |  | 1 | 1 | 0 | 1 | 1 | 1 | 1 | 0 | 0 | 0 | 0 | 1 | 1 | 1 | 1 | 1 | 1 | 0 | 1 | 1 | 2 | 1 | 1 | 2 | 0 | 0 | 1 | 0 | 1 | 2 | 0 | 2 |
|  |  | 1 | 1 | 0 | 1 | 1 | 1 | 1 | 0 | 0 | 0 | 0 | 1 | 1 | 1 | 1 | 1 | 1 | 0 | 1 | 1 | 2 | 1 | 1 | 2 | 0 | 0 | 1 | 0 | 1 | 2 | 0 | 2 |
|  |  | 1 | 1 | 0 | 1 | 1 | 1 | 1 | 0 | 0 | 0 | 0 | 1 | 1 | 1 | 1 | 1 | 1 | 0 | 1 | 1 | 2 | 1 | 1 | 2 | 0 | 0 | 1 | 0 | 1 | 2 | 0 | 2 |
|  |  | 1 | 1 | 0 | 1 | 1 | 1 | 1 | 0 | 0 | 0 | 0 | 1 | 1 | 1 | 1 | 1 | 1 | 0 | 1 | 1 | 2 | 1 | 1 | 2 | 0 | 0 | 1 | 0 | 1 | 2 | 0 | 2 |
| J.E. García-Gallo | 2018 | 1 | 1 | 0 | 1 | 1 | 1 | 1 | 1 | 0 | 1 | 1 | 1 | 1 | 1 | 1 | 1 | 1 | 0 | 1 | 1 | 1 | 1 | 1 | 1 | 1 | 0 | 1 | 0 | 1 | 0 | 0 | 0 |
| Dee W. Ford, MD, MSCR | 2016 | 2 | 1 | 2 | 0 | 2 | 1 | 0 | 1 | 0 | 0 | 0 | 1 | 1 | 1 | 1 | 1 | 1 | 0 | 1 | 1 | 1 | 1 | 1 | 2 | 1 | 0 | 1 | 0 | 1 | 2 | 0 | 2 |
| Mengshi Chen, MD | 2017 | 2 | 1 | 2 | 0 | 2 | 1 | 0 | 1 | 0 | 0 | 0 | 1 | 1 | 1 | 1 | 1 | 1 | 0 | 1 | 1 | 1 | 1 | 1 | 2 | 1 | 0 | 1 | 0 | 1 | 2 | 0 | 2 |
| Hsiao-Yun Chao | 2022 | 1 | 1 | 0 | 1 | 1 | 1 | 1 | 1 | 0 | 1 | 1 | 1 | 1 | 1 | 1 | 1 | 1 | 0 | 1 | 1 | 2 | 1 | 1 | 2 | 1 | 0 | 1 | 0 | 1 | 2 | 1 | 2 |
|  |  | 1 | 1 | 0 | 1 | 1 | 1 | 1 | 1 | 0 | 1 | 1 | 1 | 1 | 1 | 1 | 1 | 1 | 0 | 1 | 1 | 2 | 1 | 1 | 2 | 1 | 0 | 1 | 0 | 1 | 2 | 1 | 2 |
|  |  | 1 | 1 | 0 | 1 | 1 | 1 | 1 | 1 | 0 | 1 | 1 | 1 | 1 | 1 | 1 | 1 | 1 | 0 | 1 | 1 | 2 | 1 | 1 | 2 | 1 | 0 | 1 | 0 | 1 | 2 | 1 | 2 |
|  |  | 1 | 1 | 0 | 1 | 1 | 1 | 1 | 1 | 0 | 1 | 1 | 1 | 1 | 1 | 1 | 1 | 1 | 0 | 1 | 1 | 2 | 1 | 1 | 2 | 1 | 0 | 1 | 0 | 1 | 2 | 1 | 2 |
|  |  | 1 | 1 | 0 | 1 | 1 | 1 | 1 | 1 | 0 | 1 | 1 | 1 | 1 | 1 | 1 | 1 | 1 | 0 | 1 | 1 | 2 | 1 | 1 | 2 | 1 | 0 | 1 | 0 | 1 | 2 | 1 | 2 |
|  |  | 1 | 1 | 0 | 1 | 1 | 1 | 1 | 1 | 0 | 1 | 1 | 1 | 1 | 1 | 1 | 1 | 1 | 0 | 1 | 1 | 2 | 1 | 1 | 2 | 1 | 0 | 1 | 0 | 1 | 2 | 1 | 2 |
|  |  | 1 | 1 | 0 | 1 | 1 | 1 | 1 | 1 | 0 | 1 | 1 | 1 | 1 | 1 | 1 | 1 | 1 | 0 | 1 | 1 | 2 | 1 | 1 | 2 | 1 | 0 | 1 | 0 | 1 | 2 | 1 | 2 |
|  |  | 1 | 1 | 0 | 1 | 1 | 1 | 1 | 1 | 0 | 1 | 1 | 1 | 1 | 1 | 1 | 1 | 1 | 0 | 1 | 1 | 2 | 1 | 1 | 2 | 1 | 0 | 1 | 0 | 1 | 2 | 1 | 2 |
| Gary S. Phillips, MAS | 2015 | 2 | 1 | 2 | 0 | 2 | 1 | 0 | 1 | 0 | 0 | 0 | 1 | 1 | 1 | 1 | 1 | 1 | 0 | 1 | 1 | 0 | 1 | 1 | 1 | 0 | 0 | 1 | 0 | 1 | 0 | 0 | 0 |
| Xi Guo | 2022 | 2 | 1 | 2 | 0 | 2 | 1 | 0 | 1 | 0 | 0 | 0 | 1 | 1 | 1 | 1 | 1 | 1 | 0 | 1 | 1 | 2 | 1 | 1 | 2 | 0 | 0 | 1 | 0 | 1 | 2 | 0 | 2 |
|  |  | 2 | 1 | 2 | 0 | 2 | 1 | 0 | 1 | 0 | 0 | 0 | 1 | 1 | 1 | 1 | 1 | 1 | 0 | 1 | 1 | 2 | 1 | 1 | 2 | 0 | 0 | 1 | 0 | 1 | 2 | 0 | 2 |
| Minghui Gong | 2022 | 1 | 1 | 0 | 1 | 1 | 1 | 1 | 1 | 0 | 1 | 1 | 1 | 1 | 1 | 1 | 1 | 1 | 0 | 1 | 1 | 1 | 1 | 1 | 2 | 0 | 0 | 1 | 1 | 1 | 2 | 0 | 2 |
|  |  | 1 | 1 | 0 | 1 | 1 | 1 | 1 | 1 | 0 | 1 | 1 | 1 | 1 | 1 | 1 | 1 | 1 | 0 | 1 | 1 | 1 | 1 | 1 | 2 | 0 | 0 | 1 | 1 | 1 | 2 | 0 | 2 |
|  |  | 1 | 1 | 0 | 1 | 1 | 1 | 1 | 1 | 0 | 1 | 1 | 1 | 1 | 1 | 1 | 1 | 1 | 0 | 1 | 1 | 1 | 1 | 1 | 2 | 0 | 0 | 1 | 1 | 1 | 2 | 0 | 2 |
|  |  | 1 | 1 | 0 | 1 | 1 | 1 | 1 | 1 | 0 | 1 | 1 | 1 | 1 | 1 | 1 | 1 | 1 | 0 | 1 | 1 | 1 | 1 | 1 | 2 | 0 | 0 | 1 | 1 | 1 | 2 | 0 | 2 |
|  |  | 1 | 1 | 0 | 1 | 1 | 1 | 1 | 1 | 0 | 1 | 1 | 1 | 1 | 1 | 1 | 1 | 1 | 0 | 1 | 1 | 1 | 1 | 1 | 2 | 0 | 0 | 1 | 1 | 1 | 2 | 0 | 2 |
| Javier E. Garcí a-Gallo | 2019 | 1 | 1 | 0 | 1 | 1 | 1 | 1 | 1 | 0 | 1 | 1 | 1 | 1 | 1 | 1 | 1 | 1 | 0 | 1 | 1 | 1 | 1 | 1 | 2 | 0 | 0 | 1 | 0 | 1 | 2 | 0 | 2 |
| Xianfei Ding | 2022 | 2 | 1 | 2 | 0 | 2 | 1 | 0 | 1 | 0 | 0 | 0 | 1 | 1 | 1 | 1 | 1 | 1 | 0 | 1 | 1 | 2 | 1 | 1 | 2 | 1 | 0 | 1 | 0 | 1 | 2 | 1 | 2 |
|  |  | 2 | 1 | 2 | 0 | 2 | 1 | 0 | 1 | 0 | 0 | 0 | 1 | 1 | 1 | 1 | 1 | 1 | 0 | 1 | 1 | 2 | 1 | 1 | 2 | 1 | 0 | 1 | 0 | 1 | 2 | 1 | 2 |
|  |  | 2 | 1 | 2 | 0 | 2 | 1 | 0 | 1 | 0 | 0 | 0 | 1 | 1 | 1 | 1 | 1 | 1 | 0 | 1 | 1 | 2 | 1 | 1 | 2 | 1 | 0 | 1 | 0 | 1 | 2 | 1 | 2 |
| Wenxin Wang | 2021 | 2 | 1 | 2 | 0 | 2 | 1 | 0 | 1 | 0 | 0 | 0 | 1 | 1 | 1 | 1 | 1 | 1 | 0 | 1 | 1 | 2 | 1 | 1 | 2 | 1 | 0 | 1 | 0 | 1 | 2 | 1 | 2 |
| Lifeng Wang | 2021 | 1 | 1 | 0 | 1 | 1 | 1 | 1 | 1 | 0 | 1 | 1 | 1 | 1 | 1 | 1 | 1 | 1 | 0 | 1 | 1 | 2 | 1 | 1 | 2 | 1 | 0 | 1 | 0 | 1 | 2 | 1 | 2 |
| Mehtap Selcuk | 2022 | 2 | 1 | 2 | 0 | 2 | 1 | 0 | 1 | 0 | 0 | 0 | 1 | 1 | 1 | 1 | 1 | 1 | 0 | 1 | 1 | 2 | 1 | 1 | 1 | 0 | 0 | 1 | 0 | 1 | 2 | 0 | 2 |
|  |  | 2 | 1 | 2 | 0 | 2 | 1 | 0 | 1 | 0 | 0 | 0 | 1 | 1 | 1 | 1 | 1 | 1 | 0 | 1 | 1 | 2 | 1 | 1 | 1 | 0 | 0 | 1 | 0 | 1 | 2 | 0 | 2 |
|  |  | 2 | 1 | 2 | 0 | 2 | 1 | 0 | 1 | 0 | 0 | 0 | 1 | 1 | 1 | 1 | 1 | 1 | 0 | 1 | 1 | 2 | 1 | 1 | 1 | 0 | 0 | 1 | 0 | 1 | 2 | 0 | 2 |
|  |  | 2 | 1 | 2 | 0 | 2 | 1 | 0 | 1 | 0 | 0 | 0 | 1 | 1 | 1 | 1 | 1 | 1 | 0 | 1 | 1 | 2 | 1 | 1 | 1 | 0 | 0 | 1 | 0 | 1 | 2 | 0 | 2 |
|  |  | 2 | 1 | 2 | 0 | 2 | 1 | 0 | 1 | 0 | 0 | 0 | 1 | 1 | 1 | 1 | 1 | 1 | 0 | 1 | 1 | 2 | 1 | 1 | 1 | 0 | 0 | 1 | 0 | 1 | 2 | 0 | 2 |
|  |  | 2 | 1 | 2 | 0 | 2 | 1 | 0 | 1 | 0 | 0 | 0 | 1 | 1 | 1 | 1 | 1 | 1 | 0 | 1 | 1 | 2 | 1 | 1 | 1 | 0 | 0 | 1 | 0 | 1 | 2 | 0 | 2 |
|  |  | 2 | 1 | 2 | 0 | 2 | 1 | 0 | 1 | 0 | 0 | 0 | 1 | 1 | 1 | 1 | 1 | 1 | 0 | 1 | 1 | 2 | 1 | 1 | 1 | 0 | 0 | 1 | 0 | 1 | 2 | 0 | 2 |
|  |  | 2 | 1 | 2 | 0 | 2 | 1 | 0 | 1 | 0 | 0 | 0 | 1 | 1 | 1 | 1 | 1 | 1 | 0 | 1 | 1 | 2 | 1 | 1 | 1 | 0 | 0 | 1 | 0 | 1 | 2 | 0 | 2 |
| Vicent J. Ribas | 2018 | 1 | 1 | 0 | 1 | 1 | 1 | 1 | 1 | 0 | 1 | 1 | 1 | 1 | 1 | 1 | 1 | 0 | 0 | 0 | 0 | 2 | 1 | 1 | 2 | 0 | 0 | 1 | 0 | 1 | 2 | 0 | 2 |

**Table S4** Modelling variables.

| No | Death within 1 month | Fre. | Death in hospital | Fre. | Best model | RF | XGBoost | All models | Fre. |
| --- | --- | --- | --- | --- | --- | --- | --- | --- | --- |
|  | variables |  | variables |  | variables |  |  | variables |  |
| 1 | Age | 14 | GCS | 28 | Age | 9 | 7 | Lactate | 46 |
| 2 | Lactate | 13 | Age | 27 | Lactate | 7 | 6 | Age | 45 |
| 3 | Heartrate | 12 | Lactate | 27 | Systolic blood pressure | 7 | 6 | GCS | 37 |
| 4 | Shock | 12 | pH | 26 | Heartrate | 7 | 6 | Systolic blood pressure | 34 |
| 5 | Calcium | 11 | Gender | 24 | GCS | 7 | 4 | pH | 33 |
| 6 | SpO2 | 11 | Ventilator | 23 | SpO2 | 6 | 5 | Ventilator | 31 |
| 7 | BE | 11 | Systolic blood pressure | 21 | Temperature | 6 | 5 | Respiratory rate | 31 |
| 8 | Systolic blood pressure | 10 | Respiratory rate | 19 | BUN | 6 | 4 | Heartrate | 30 |
| 9 | Albumin | 10 | PTT | 18 | Ventilator | 6 | 4 | Temperature | 30 |
| 10 | GCS | 9 | PaO2 | 18 | Respiratory rate | 7 | 3 | SpO2 | 27 |
| 11 | Respiratory rate | 9 | BUN | 17 | Creatinine | 5 | 4 | Gender | 26 |
| 12 | Cancer | 9 | Comorbidities | 17 | pH | 6 | 3 | BUN | 26 |
| 13 | Temperature | 9 | Urine output | 16 | PLT | 4 | 4 | Creatinine | 24 |
| 14 | PLT | 9 | Temperature | 16 | Urine output | 4 | 3 | PaO2 | 22 |
| 15 | SOFA score | 8 | BMI | 16 | INR | 4 | 3 | INR | 21 |
| 16 | BUN | 8 | PCO2 | 15 | PaO2 | 4 | 3 | PCO2 | 21 |
| 17 | RDW | 8 | Heartrate | 14 | TBIL | 3 | 3 | PLT | 21 |
| 18 | INR | 8 | Creatinine | 13 | SOFA score | 4 | 2 | SOFA score | 20 |
| 19 | Total CO2 | 8 | SpO2 | 13 | Calcium | 3 | 3 | Urine output | 20 |
| 20 | Creatinine | 7 | PT | 13 | Cancer | 3 | 3 | PTT | 19 |
| 21 | pH | 7 | INR | 13 | Mean blood pressure | 5 | 1 | WBC | 19 |
| 22 | Bicarbonate | 7 | Mean blood pressure | 13 | Total CO2 | 4 | 2 | Shock | 19 |
| 23 | IL8 | 7 | WBC | 13 | WBC | 3 | 3 | Comorbidities | 19 |
| 24 | PCT | 7 | Bacterial infections | 13 | Gender | 3 | 2 | Cancer | 18 |
| 25 | IL-6 | 7 | Cancer | 12 | AG | 3 | 2 | Mean blood pressure | 18 |
| 26 | Ventilator | 6 | Blood cultures and antibiotics | 12 | PTT | 4 | 1 | Albumin | 17 |
| 27 | PCO2 | 6 | AG | 11 | BE | 3 | 2 | BMI | 17 |
| 28 | FIO2 | 6 | Race | 11 | Albumin | 3 | 2 | BE | 16 |
| 29 | D-dimer | 6 | Chronic disease | 11 | PCO2 | 3 | 2 | Bicarbonate | 15 |
| 30 | Race | 4 | Blood transfusion | 11 | BMI | 2 | 3 | Total CO2 | 15 |
| 31 | WBC | 4 | Dobutamine | 11 | FIO2 | 2 | 3 | Race | 15 |
| 32 | Urine output | 3 | HCT | 11 | Shock | 3 | 2 | PT | 14 |
| 33 | AG | 3 | Renal failure | 11 | Comorbidities | 2 | 3 | AG | 14 |
| 34 | Chronic disease | 3 | Shock | 11 | Vasopressor | 3 | 1 | Chronic disease | 14 |
| 35 | NLR | 2 | Insurance types | 10 | PT | 3 | 1 | TBIL | 13 |
| 36 | PLR | 2 | Marital status | 9 | Unit | 3 | 1 | Calcium | 13 |
| 37 | LMR | 2 | Languages | 9 | Mean diastolic BP | 2 | 2 | Bacterial infections | 13 |
| 38 | Vasopressor | 2 | SOFA score | 9 | Bicarbonate | 3 | 1 | Blood cultures and antibiotics | 12 |
| 39 | Unit | 2 | Potassium | 9 | Bacterial infections | 2 | 2 | RDW | 11 |
| 40 | Sodium | 2 | BE | 8 | Potassium | 3 |  | Blood transfusion | 11 |
| 41 | Respiratory failure | 2 | Bicarbonate | 8 | RDW | 3 |  | Dobutamine | 11 |
| 42 | DFA(a2) | 2 | Anemia | 8 | Metastatic solid tumor | 2 | 1 | HCT | 11 |
| 43 | TBIL | 1 | Digestive disorders | 8 | Race | 2 | 1 | Renal failure | 11 |
| 44 | PT | 1 | PLT | 8 | PaO2/FiO2 | 2 | 1 | Insurance types | 10 |
| 45 | AST | 1 | Fever | 8 | Respiratory failure | 2 | 1 | Vasopressor | 10 |
| 46 | PTT | 1 | TBIL | 7 | Cardiac insufficiency | 1 | 2 | Mean diastolic BP | 10 |
| 47 | MCHC | 1 | RDW | 7 | Chronic disease | 2 | 1 | FIO2 | 10 |
| 48 | Mean blood pressure | 1 | Total CO2 | 7 | Glucose | 1 | 2 | Marital status | 9 |
| 49 | Metastatic solid tumor | 1 | Respiratory failure | 7 | CRP | 1 | 2 | Languages | 9 |
| 50 | PaO2 | 1 | CRP | 7 | Hemoglobin | 1 | 2 | Potassium | 9 |
| 51 | Thrombomodulin | 1 | PEEP | 7 | PCT | 2 | 1 | Unit | 9 |
| 52 | Peritonitis no surgery | 1 | Unit | 6 | IL-6 | 2 | 1 | PaO2/FiO2 | 9 |
| 53 | Cardiac insufficiency | 1 | Albumin | 6 | CVP | 2 | 1 | Sodium | 8 |
| 54 | Consciousness disturbance | 1 | Metastatic solid tumor | 6 | Blood transfusion | 2 | 1 | Anemia | 8 |
| 55 | APACHE-III | 1 | Mean diastolic BP | 6 | Dobutamine | 2 | 1 | Digestive disorders | 8 |
| 56 | Charlson comorbidity index | 1 | PaO2/FiO2 | 6 | Blood cultures and antibiotics | 2 | 1 | Respiratory failure | 8 |
| 57 | CCL3 | 1 | Sodium | 6 | HCT | 2 | 1 | Cardiac insufficiency | 8 |
| 58 | HSPA1B | 1 | Maximum chloride | 6 | Renal failure | 2 | 1 | CRP | 8 |
| 59 | GZMB | 1 | Cardiac insufficiency | 6 | Fever | 2 | 1 | Fever | 8 |
| 60 | CCL4 | 1 | Urinary tract infections | 6 | AST | 2 |  | Metastatic solid tumor | 7 |
| 61 | Blood group | 1 | Vasopressor | 5 | Sodium | 1 | 1 | IL8 | 7 |
| 62 | Magnesium | 1 | AST | 4 | Anemia | 1 | 1 | PCT | 7 |
| 63 | Glucose | 1 | Charlson comorbidity index | 4 | Digestive disorders | 1 | 1 | IL-6 | 7 |
| 64 | CRP | 1 | Glucose | 4 | IL8 | 1 | 1 | CVP | 7 |
| 65 | ALAT | 1 | Hemoglobin | 4 | Urinary tract infections | 1 | 1 | PEEP | 7 |
| 66 | AFib in history | 1 | CVP | 4 | PEEP | 1 | 1 | D-dimer | 7 |
| 67 | Sodium | 1 | Intravenous fluids | 4 | D-dimer | 1 | 1 | Maximum chloride | 6 |
| 68 | Lipase | 1 | Cardiovascular score | 4 | Marital status | 1 |  | Glucose | 6 |
| 69 | r-GT | 1 | HRnV | 4 | Insurance types | 1 |  | Hemoglobin | 6 |
| 70 | Alkaline phosphatase | 1 | Beta-blocker | 3 | Languages | 1 |  | Urinary tract infections | 6 |
| 71 | Hemoglobin | 1 | Cardiac dysrhythmia | 3 | Phosphate | 1 |  | AST | 5 |
| 72 | Mean NN (s) | 1 | ECG performed | 3 | MCHC | 1 |  | Charlson comorbidity index | 5 |
| 73 | SDNN | 1 | Phosphate | 2 | Maximum chloride | 1 |  | HRnV | 5 |
| 74 | LF/HF | 1 | Calcium | 2 | Charlson comorbidity index | 1 |  | Intravenous fluids | 4 |
| 75 | HRnV | 1 | Coagulopathy | 2 | Beta-blocker | 1 |  | Cardiovascular score | 4 |
| 76 | Chills | 1 | Peripheral vascular diseases | 2 | Cardiac dysrhythmia | 1 |  | NLR | 3 |
| 77 | Fever | 1 | Neurosurgery | 2 | ECG performed | 1 |  | Beta-blocker | 3 |
| 78 | The thrombodynamic ratio | 1 | CRRT | 2 | Blood group |  | 1 | Cardiac dysrhythmia | 3 |
| 85 | Mean diastolic BP | 0 | Liver disease | 1 | Alkaline phosphatase |  | 1 | Neurosurgery | 2 |
| 86 | PaO2/FiO2 | 0 | Organ transplanted | 1 | Intravenous fluids | 1 |  | Magnesium | 2 |
| 87 | Maximum chloride | 0 | TnT | 1 | Cardiovascular score | 1 |  | DFA (a2) | 2 |
| 88 | Anemia | 0 | Chills | 1 | HRnV |  | 1 | CRRT | 2 |
| 89 | Digestive disorders | 0 | suPAR | 1 | Chills | 1 |  | MCHC | 1 |
| 90 | Coagulopathy | 0 | PLR | 0 | NLR |  |  | Thrombomodulin | 1 |
| 91 | Peripheral vascular diseases | 0 | LMR | 0 | PLR |  |  | Peritonitis no surgery | 1 |
| 92 | Neurosurgery | 0 | MCHC | 0 | LMR |  |  | Consciousness disturbance | 1 |
| 93 | BMI | 0 | Thrombomodulin | 0 | Thrombomodulin |  |  | APACHE-III | 1 |
| 94 | Beta-blocker | 0 | Peritonitis no surgery | 0 | Coagulopathy |  |  | CCL3 | 1 |
| 95 | Cardiac dysrhythmia | 0 | Consciousness disturbance | 0 | Peripheral vascular diseases |  |  | HSPA1B | 1 |
| 96 | ECG performed | 0 | APACHE-III | 0 | Neurosurgery |  |  | GZMB | 1 |
| 97 | CVP | 0 | CCL3 | 0 | Peritonitis no surgery |  |  | CCL4 | 1 |
| 98 | Antimicrobial therapy time | 0 | HSPA1B | 0 | Consciousness disturbance |  |  | Blood group | 1 |
| 99 | APACHE II | 0 | IL8 | 0 | APACHE-III |  |  | ALAT | 1 |
| 100 | Intravenous fluids | 0 | GZMB | 0 | CCL3 |  |  | AFib in history | 1 |
| 101 | Blood transfusion | 0 | CCL4 | 0 | HSPA1B |  |  | Sodium | 1 |
| 102 | Dobutamine | 0 | Blood group | 0 | GZMB |  |  | Lipase | 1 |
| 103 | Blood cultures and antibiotics | 0 | ALAT | 0 | CCL4 |  |  | r-GT | 1 |
| 104 | Cardiovascular score | 0 | AFib in history | 0 | Antimicrobial therapy time |  |  | Alkaline phosphatase | 1 |
| 105 | HCT | 0 | Sodium | 0 | APACHE II |  |  | Antimicrobial therapy time | 1 |
| 106 | Renal failure | 0 | Lipase | 0 | Mean NN (s) |  |  | APACHE II | 1 |
| 107 | Liver disease | 0 | r-GT | 0 | DFA (a2) |  |  | Mean NN (s) | 1 |
| 108 | Organ transplanted | 0 | Alkaline phosphatase | 0 | Liver disease |  |  | Liver disease | 1 |
| 109 | CRRT | 0 | PCT | 0 | Organ transplanted |  |  | Organ transplanted | 1 |
| 110 | TnT | 0 | IL-6 | 0 | CRRT |  |  | TnT | 1 |
| 111 | Urinary tract infections | 0 | FIO2 | 0 | TnT |  |  | SDNN | 1 |
| 112 | Bacterial infections | 0 | SDNN | 0 | SDNN |  |  | LF/HF | 1 |
| 113 | Comorbidities | 0 | LF/HF | 0 | LF/HF |  |  | Chills | 1 |
| 114 | PEEP | 0 | D-dimer | 0 | BNP |  |  | BNP | 1 |
| 115 | BNP | 0 | BNP | 0 | The thrombodynamic ratio |  |  | The thrombodynamic ratio | 1 |
| 116 | suPAR | 0 | The thrombodynamic ratio | 0 | suPAR |  |  | suPAR | 1 |

Notes: Fre. indicates the frequency of modeling variables.
